# Fixed‐Linkage Enabled Ultra‐Stable Ion Transport Channels in Membranes for Long‐Life Alkaline Flow Batteries

**DOI:** 10.1002/advs.202524374

**Published:** 2026-04-14

**Authors:** Zhiquan Wei, Jiaxiong Zhu, Yiqiao Wang, Xinru Yang, Dedi Li, Hong Hu, Qingshun Nian, Shaoce Zhang, Shixun Wang, Zhuoxi Wu, Yue Hou, Shengnan Wang, Ze Chen, Qing Li, Chunyi Zhi

**Affiliations:** ^1^ Department of Materials Science and Engineering City University of Hong Kong Hong Kong China; ^2^ Hong Kong Center for Cerebro‐Cardiovascular Health Engineering (COCHE) Hong Kong China; ^3^ School of Interdisciplinary Studies Lingnan University Hong Kong China; ^4^ Institute of Applied Physics and Materials Engineering University of Macau Macau SAR China; ^5^ Materials Innovation Institute for Life Sciences and Energy (MILES) The University of Hong Kong‐Shenzhen Institute of Research and Innovation (HKU‐SIRI) Shenzhen China; ^6^ Center for Energy Storage The University of Hong Kong Hong Kong China; ^7^ Department of Mechanical Engineering The University of Hong Kong Hong Kong China

**Keywords:** alkaline flow batteries, fixed linkages‐based polymer, high‐flux ion transport channels, ultra‐stable membrane

## Abstract

Alkaline zinc–iron redox flow batteries (Zn─Fe FBs) are promising candidates for developing high‐voltage and cost‐effective grid‐scale energy storage systems. However, the low ionic conductivity and poor alkaline stability of membranes lead to battery performance degradation and shortened lifespan. Here, we develop fixed ethylene linkage‐based polybenzimidazole (FE‐PBI) membranes to break this dilemma. Introducing fixed ethylene linkages enlarges the ion transport channel and provides inter‐support for polymer chains, thereby preserving highly ordered and flux ion transport channels in the alkaline environment. By this unique channel design, this FE‐PBI membrane enables alkaline Zn‐based asymmetric FBs to deliver excellent rate performance (up to 240 mA cm^−2^) with high reversibility of Zn deposition. Furthermore, the Zn─Fe FBs using FE‐PBI membranes deliver a highly stable rate performance across the range of 60–180 mA cm^−2^ and 120 mAh cm^−2^. At a challenging condition of high current density (100 mA cm^−2^) and areal capacity (80 mAh cm^−2^), the developed Zn─Fe FBs achieve an impressive energy efficiency of >85.56% and remarkable stability over 800 h (500 cycles), surpassing the lifespan performance of current alkaline Zn─Fe FBs. This work offers a new membrane design approach to combine high energy efficiency with an extended lifespan, realizing long‐lifetime alkaline FBs.

## Graphical abstract

Rigid fixed‐ethylene‐linked PBI membrane stabilizes ion‐transport channels, enabling high ionic conductivity and durable alkaline Zn─Fe flow batteries.

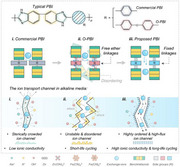

## 1 Introduction

**FIGURE 1:**
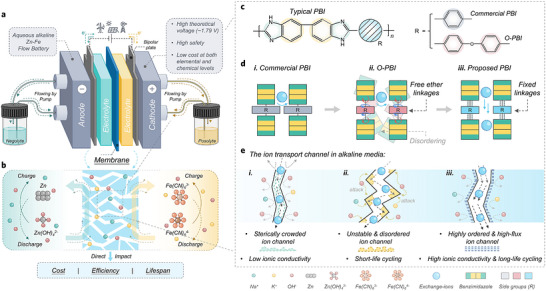
Schematic illustrations showing existing and proposed ion‐selective polymer membranes with highly ordered and high‐flux ion channels. (a,b) A schematic diagram of alkaline Zn─Fe FBs with the relevant chemical reactions. (c), Chemical structure of typical PBI polymers. (d,e) Schematic showing commercial PBI (d (i), e (i)), mainstream‐developed O‐PBI (d (ii),e (ii)), and proposed PBI membrane (d (iii),e (iii)) with corresponding issues and potentials for the ion transferring channels in alkaline media.

The integration of intermittent renewable energy into power grids critically depends on robust energy storage systems that prioritize operational safety and extended longevity [1, 2, 3]. Aqueous redox flow batteries (FBs) stand out as a transformative technology for stationary storage, leveraging their unique architecture in decoupled power and energy capabilities [4, 5, 6, 7, 8, 9, 10, 11]. Among those options, alkaline zinc–iron (Zn─Fe) FBs emerge as a compelling alternative to commercial vanadium (V) FBs due to their high theoretical voltage (∼1.79 vs. ∼1.26V), high safety with low toxicity (alkaline vs. corrosive acidic sulfuric system), and cost‐effectiveness of electrolytes at chemical/element levels ($ 0.64 L^−1^ vs. $ 3.7–7.1 L^−1^) (Figure 1a) [12, 13, 14].

The membrane is a critical component in alkaline Zn─Fe FBs, playing a pivotal role in determining their overall cost, efficiency, and lifespan (Figure 1b) [15, 16, 17, 18]. However, the current membranes still suffer from a short operating lifetime in alkaline Zn─Fe FBs, failing to meet the widespread application requirements for energy storage systems due to unresolved challenges related to the instability of membranes, side reaction of Zn metal anode, and irreversible crossover issues [19, 20, 21, 22, 23]. Taking commercially available Nafion membranes based on perfluorosulfonic acid (PFSA) materials as an example, their high cost (>$ 500 m^−2^), sluggish ionic conductivity, and environmental concerns pose significant limitations, leading to low efficiency of alkaline Zn─Fe FBs [13, 24, 25]. To overcome these limitations, polybenzimidazole (PBI), another commercially available polymer membrane, has emerged as a promising alternative due to its low cost (< $ 10 m^−2^) and relatively high chemical stability in both acidic/alkaline electrolytes (Figure 1c) [26, 27, 28, 29, 30]. However, despite those advantages, the bottleneck of PBI membranes remains the low ionic conductivity, which is caused by sterically crowded ionic channels resulting from intrinsically short polymer chains (Figure 1d‐i, e‐i) [31].

Most research focuses on extending the steric chain length of the PBI polymer structure by introducing ether linkages (O‐PBI, Figure 1c), which enlarges the size of the ion transport channels, thereby enhancing the ionic conductivity (Figure 1d‐ii) [29, 32, 33]. Unfortunately, the free ether linkages of O‐PBI may increase the tendency of the polymer chains to interlock or stack during cycling in alkaline Zn─Fe FBs (Figure 1d‐ii,e‐ii), disrupting the ion transport channels, reducing membrane stability, and ultimately shortening battery lifespan [34]. Despite efforts to enhance the stability of O‐PBI membranes in alkaline media through the pre‐coordination of various metal ions (i.e., Cu^2+^, Zn^2+^, Co^2+^), failing to fundamentally resolve the inherent challenges posed by the free ether linkages remains a major barrier for further development [12, 34, 35]. In other words, if fixed‐rigid centers are anchored within polymer chain segments to restrict the motion of free‐linkage units (Figure 1d‐iii), this molecular structure design could enforce a dynamically stable and high‐flux ion transport channel for long‐chain polymers. Therefore, such a membrane enabling highly ordered ion highways could maintain robust and stable ionic conductivity throughout prolonged cycling (Figure 1e‐iii), holding significant potential to ensure high efficiency with exceptional operational longevity in alkaline Zn─Fe FBs.

Here, we develop a novel fixed ethylene linkages‐based PBI (FE‐PBI) polymer that provides a rigid ionic highway and strong alkalinity resistance. Molecular dynamics simulations and experimental analyses indicate that these rigid ion transport channels facilitate inter‐support between FE‐PBI polymers and reduce the metal ion coordination, contributing to a high persistence in alkaline media. As a result, endowed by the fast transport of charge carriers within this membrane, alkaline Zn‐based asymmetric FBs demonstrate a highly reversible Zn metal plating/stripping process at a high current density level, exhibiting excellent rate performance with a long lifespan. The developed alkaline Zn─Fe FBs using FE‐PBI membranes deliver high power density and stable rate‐performance at an areal capacity of 120 mAh cm^−2^. Furthermore, FE‐PBI membranes demonstrate a favorable combination of conductivity and stability, achieving a high energy efficiency of over 85.56% and an impressively long life of over 800 h at a current density of 100 mA cm^−2^ in alkaline Zn─Fe FBs. This work offers a novel pathway for the rational design of membranes tailored to sustainable energy storage technologies, including safe, eco‐friendly, and high‐performance FB systems.

## 2 Results

### 2.1 Enhanced Transport Properties

**FIGURE 2:**
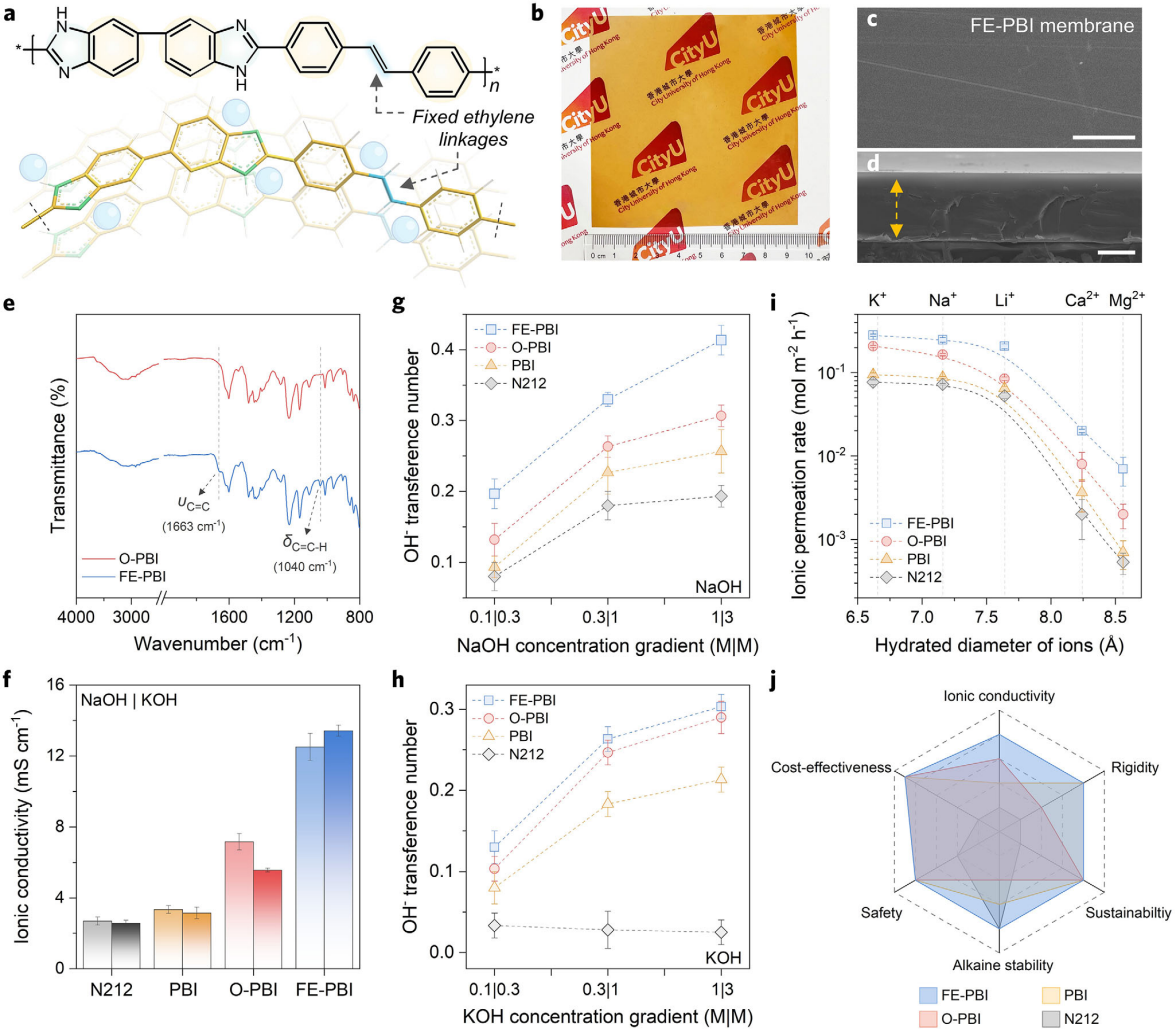
Structure and properties of FE‐PBI membrane in alkaline media. (a) Monomer molecular structures of FE‐PBI with fixed‐ethylene linkages. (b) Digital image of free‐standing FE‐PBI membrane with a size of over 9 cm. (c,d) Surface and cross‐sectional SEM image of FE‐PBI membrane. The corresponding scale bars are 25 and 20 µm. (e) FTIR spectra of O‐PBI and FE‐PBI membranes. (f) Ionic conductivity of N212, PBI, O‐PBI, and FE‐PBI membranes in 1 m NaOH and 1 m KOH solution. (g,h) The OH^−^ (t^−^) ion transference number of N212, PBI, O‐PBI, and FE‐PBI membranes for different alkaline (NaOH and KOH) concentration gradients. (i) Selective ion permeation of common salts through N212, PBI, O‐PBI, and FE‐PBI membranes. (j) Qualitative ranking of key performance indicators for N212, PBI, O‐PBI, and FE‐PBI membranes. The key parameters are ranked on a scale from 1 to 5.

Considering that a highly rigid main chain structure would hinder polymer membrane formation, ethylene (carbon‐carbon double bonds, ─C═C─) is chosen as the fixed linkage center for constructing the proposed FE‐PBI‐based polymer membrane, denoted as FE‐PBI in the following text [36, 37, 38, 39]. A gradient‐heating dehydration condensation reaction was adopted for the synthesis (Figure 2a; Figure S1), and a solution casting method with low‐temperature drying was employed to obtain smooth, fully transparent, and homogeneous ion‐exchange membranes (Figure 2b) [28]. Furthermore, the uniformity of the membranes was confirmed by the surface and cross‐sectional scanning electron microscopy (SEM) images (Figure 2c,d). The molecular architecture was synergistically validated through multi‐spectral characterization. Fourier‐transform infrared spectroscopy (FTIR) revealed diagnostic vibrations at 1663 cm^−1^ (ν_‐C = C‐_ stretching) and 1040 cm^−1^ (δ_C‐H_ in‐plane bending of conjugated diene), confirming the fixed linkage center of ─C═C─ sites (Figure 2e) [40, 41, 42]. Furthermore, the successful synthesis was further confirmed by ^1^H nuclear magnetic resonance (NMR) (Figure S2), and the superior structural thermal stability of FE‐PBI through enhanced *π*‐conjugation rigidity was demonstrated by thermogravimetric analysis (TG) (Figure S3). In light of cis‐trans isomerization, the major component is identified to be trans‐structure FE‐PBI, ensuring the formation of stable and ordered ion transport channels (Figure S4) [43].

The transport of ions across a membrane relies on the supporting electrolyte and the movement of charge carriers. The ionic conductivity of thick (35–50 µm) membranes with different polymer structures was measured using electrochemical impedance spectroscopy (EIS) under alkaline media (Figure 2f; Figure S5). The ionic conductivity of the FE‐PBI membrane reaches over 12 mS cm^−1^ in both NaOH and KOH alkaline electrolytes. In contrast, the conductivity of current membranes for alkaline Zn─Fe FBs (for example, O‐PBI, commercial PBI, and Nafion 212 (N212)) is significantly lower. This supports our hypothesis of developing highly ordered flux ion transport channels in FE‐PBI membranes.

To understand ion transport behavior in these membranes, we measured and calculated the ion transference number of cations and hydroxide (OH^−^) by collecting voltage‐current (V‐I) profiles (Figures S6 and S7) in different alkaline concentration gradients (0.1|0.3, 0.3|1, and 1|3 m). As shown in Figure 2g,h, cations (K^+^ or Na^+^) are the dominant charge carriers in the N212 membrane. In contrast, the OH^−^ transference number of the PBI‐based membranes is higher than that of the N212 membrane (O‐PBI: 0.30 in 1|3 m NaOH and 0.29 in 1|3 m KOH; PBI: 0.26 in 1|3 m NaOH and 0.21 in 1|3 m KOH; N212: 0.19 in 1|3 m NaOH and 0.03 in 1|3 m KOH). When NaOH or KOH concentration reaches 1|3 m, FE‐PBI membranes (0.41 in 1|3 m NaOH and 0.30 in 1|3 m KOH) exhibit a greater OH^−^ transport ratio than other membranes, suggesting that FE‐PBI membranes could transfer OH^−^ more effectively in higher alkaline concentration solutions. The Grottuss transport mechanism contributes to the high OH^−^ transport selectivity, resulting in high ionic conductivity for FE‐PBI membranes [13].

The ionic transport behavior in membranes was further investigated using a concentration‐driven configuration [35]. Permeability tests were conducted using 1 m aqueous metal salt solutions, including KCl, NaCl, LiCl, CaCl_2_, and MgCl_2_ as the feeding solution, with deionized water on the permeate side. As displayed in Figure 2i, FE‐PBI membranes exhibit the highest permeation rate of hydrated K^+^ ions among all tested membranes. Notably, FE‐PBI membranes exhibit a sharp size‐exclusion cut‐off of ∼8.0 Å, allowing the fast transport of smaller‐sized hydrated ions (K^+^, Na^+^, Li^+^) along with blocking the permeation of redox‐active ions‐[Fe(CN)_6_]^3−^ anions (∼9.5 Å). As a result, FE‐PBI membranes display a similar permeation rate of the [Fe(CN)_6_]^3−^ with other membranes in avoiding crossover (Figures S8–S13). This selective transport arises from the combined effects of a stable transport‐relevant nanostructure, restricted ion‐transport pathways, and a weak charge‐related exclusion contribution (Figures S14–S16).

Clearly, the as‐fabricated FE‐PBI membrane delivers the highest ionic conductivity and OH^−^ transference number among all membranes for alkaline Zn─Fe FBs systems. Moreover, a comparison of key performance indicators, including ionic conductivity, cost‐effectiveness, sustainability, alkaline stability, safety, and tensile strength, suggests that this low‐cost hydrocarbon membrane is a promising alternative to benchmark conventional PBI and Nafion membranes (Figure 2j; Figure S16, and key parameters in Table S1).

### 2.2 Dendrite‐Free Performance in High Energy Densities

**FIGURE 3:**
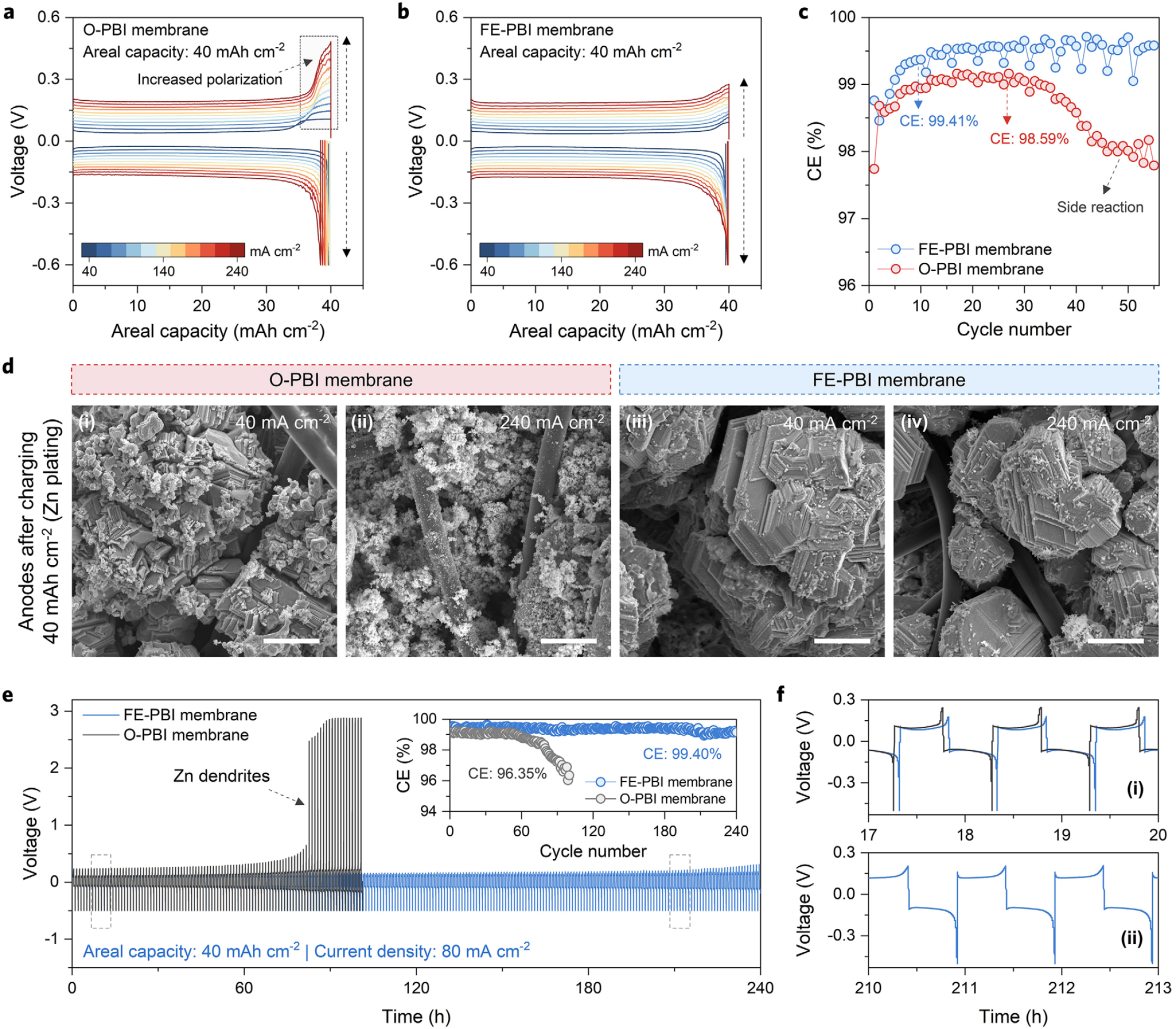
Electrochemical performance of alkaline Zn‐based asymmetric FBs. (a–c) Rate performance and corresponding CE comparison of alkaline Zn‐based asymmetric FBs assembled with O‐PBI and FE‐PBI membranes at an areal capacity of 40 mAh cm^−2^. (d) SEM images of anodes after charging (Zn plating) 40 mAh cm^−2^ with a current density of 40 and 240 mA cm^−2^ in alkaline Zn‐based asymmetric FBs assembled with O‐PBI (i, ii) and FE‐PBI membranes (iii, iv). The corresponding scale bars are 25 µm. (e) Cycling performance of alkaline Zn‐based asymmetric FBs assembled with O‐PBI and FE‐PBI membranes at an areal capacity of 40 mAh cm^−2^. The inset shows the corresponding CE comparison. (f) Detailed (i and ii) voltage profiles marked by the rectangles by cycling performance e.

The liquid/solid transition in the Zn dissolution/deposition process presents a significant challenge in Zn─Fe FBs, significantly impacting overall battery performance. Zn dendrites form in diffusion‐limited conditions, which becomes especially problematic at high areal capacities and current densities, leading to reduced reversibility and shortened cycle life [34, 44, 45]. Membranes that control the ion transport rate significantly affect the performance of the zinc anode. Motivated by this, Zn‐based asymmetrical FBs were used to investigate the influence of membranes on Zn deposition behavior systematically. As the current density increases from 40 to 240 mA cm^−2^ (Figure 3a), a significant polarization rise can be observed at the end stage of the deposition process, which is due to the limited ionic conductivity of the O‐PBI membrane. In contrast, Zn‐based asymmetrical FBs with FE‐PBI membranes can maintain stable Zn deposition voltage curves (Figure 3b) at high current densities up to 240 mA cm^−2^. Meanwhile, as shown in Figure 3c and Figure S17, Zn‐based asymmetrical FBs using FE‐PBI membranes exhibit higher Coulombic efficiency (CE, average ∼99.41%) at different current densities than those using O‐PBI membranes (average ∼98.59%). This result demonstrates that the fast charge carrier transport of FE‐PBI membranes facilitates highly efficient and reversible Zn deposition and stripping processes.

To gain insight into Zn plating throughout the charging process, we further characterized the morphologies and structures of plated Zn on the anode with different current densities. As shown in the SEM images of Figure 3d, the Zn plating morphology using O‐PBI membranes evolves from granular forms (Figure 3d‐i) to disordered porous shapes (Figure 3d‐ii) with increasing current density from 40 to 240 mA cm^−2^. This is due to the low ionic conductivity of O‐PBI membranes, which contributes to the formation of uneven zinc plating, and this heterogeneous deposition deteriorates with the increase of current density. In contrast, the Zn plating morphology using FE‐PBI membranes consistently forms ordered hexagonal plate‐like structures across the current density range (Figure 3d‐iii,d‐iv). The underlying reason is the ionic conductivity difference of the corresponding membranes (FE‐PBI > O‐PBI > commercial PBI membranes, Figure 2e,f), which leads to lower polarization and better Zn(OH)_4_^2−^ diffusion to guarantee the ordered Zn plating morphologies for FE‐PBI membranes. The Zn plating morphology utilizing a commercial PBI membrane with the lowest ionic conductivity even results in the formation of disordered granular Zn metal structures at low current densities (Figure S18). Furthermore, the corresponding X‐ray diffraction (XRD) analysis (Figure S19) also supports these results, showing that the plated Zn using FE‐PBI exhibits a preferred (002) crystal plane, promoting the formation of an ordered hexagonal plate stack‐like Zn morphology [46].

The long‐term cycling behavior of Zn‐based asymmetric FBs under a high areal current (80 mA cm^−2^, 40 mAh cm^−2^) was further used to demonstrate the robustness of the FE‐PBI membrane on the constant Zn plating/stripping process. As displayed in Figure 3e, the Zn‐based asymmetric FBs using O‐PBI membranes exhibit an inferior lifespan of approximately 100 h. In contrast, the FE‐PBI membrane enables the Zn‐based asymmetric FBs to cycle reversibly over 240 h (Figure 3e and the inset of CE). This long‐term cycle stability can be attributed to the stable and low overpotential level (Figure 3f), which is faithfully consistent with the ionic transport properties revealed in Figure 2. The above results indicate that FE‐PBI membranes with high ionic conductivity endowed by the high‐order and flux ions transport channel, can extend diffusion restrictions, induce conformal Zn morphology, and promote reversible Zn metal plating/stripping.

### 2.3 Anti‐Alkali Rigid Structure Supporting a Durable Ionic Highway

**FIGURE 4:**
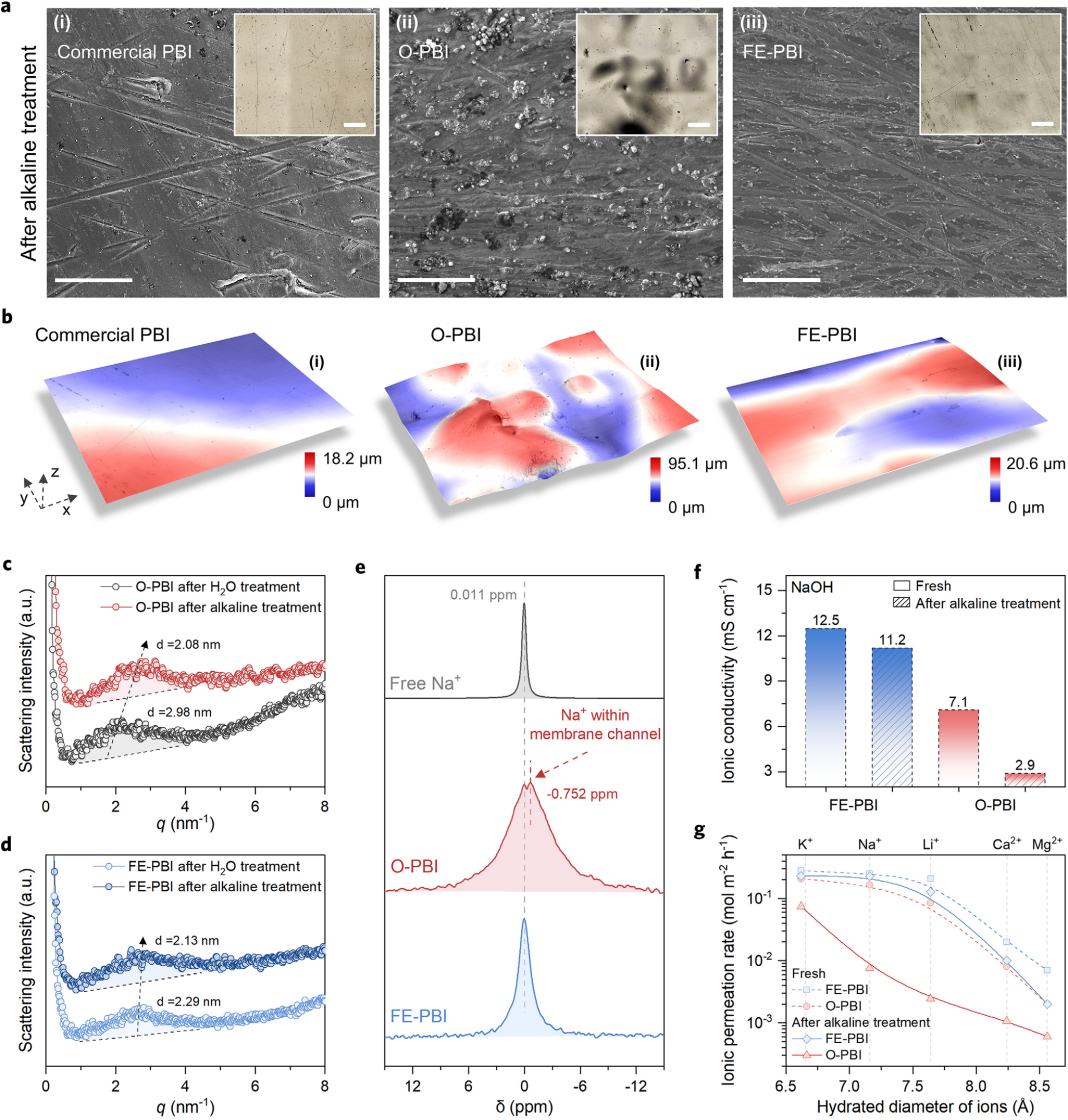
Alkali resistance properties of FE‐PBI membrane. (a,b) Surface morphology, and of (i) commercial PBI, (ii) O‐PBI, and (iii) FE‐PBI membranes after alkaline treatment, characterized by SEM, optical microscopy (insets), and laser confocal scanning microscopy. (c,d) SAXS profiles of O‐PBI and FE‐PBI membranes before/after alkaline treatment. (e) ssNMR measured for O‐PBI and FE‐PBI membranes. 0.1 m NaOH in water was used as the control, and all membranes were immersed in 0.1 m NaOH solution after testing. f Ionic conductivity of O‐PBI and FE‐PBI membranes in 1 m NaOH solution before/after alkaline treatment. g Selective ion permeation of common salts through O‐PBI and FE‐PBI membranes before/after alkaline treatment in 3.8 m NaOH.

To further explore the origin of the superior stability, we studied the alterations of the membrane aged in alkaline media (treated in a 3.8 m NaOH solution at 60°C for one month). As shown in Figure 4a, after strong alkaline treatment, the short‐chain structured membranes (Commercial PBI, Figure 4a‐i) and fixed rigid structured membranes (FE‐PBI, Figure 4a‐iii) maintain an initial appearance, while the flexible O‐PBI membrane exhibits a deformed surface (Figure 4a‐ii). Meanwhile, the results obtained from laser confocal scanning microscopy (LCSM) also verify the uneven surface of the aged O‐PBI membrane (Figure 4b‐ii), indicating its weak alkaline tolerance. In contrast, the aged FE‐PBI membrane (Figure 4b‐iii) exhibits a smooth surface similar to the aged commercial PBI (Figure 4b‐i). The small‐angle X‐ray scattering (SAXS) technique is utilized to investigate water cluster size and hydrated ion clusters in ion channels within membranes [47, 48, 49]. The 2D SAXS patterns (Figures S20 and S21) display isotropy in both membranes after alkaline treatment, showing an isotropic dispersion and orientation of heterogeneities within both polymers [50]. Specifically, O‐PBI exhibits a pronounced shift of the characteristic scattering peak from *q* = 2.1 to 3 nm^−1^ after alkaline treatment, whereas FE‐PBI shows only a much smaller shift from *q* = 2.74 to 2.95 nm^−1^ (Figure 4c,d). The corresponding characteristic domain spacing, which is associated with the organization of transport‐relevant ionic channels/domains in the membrane, decreases from 2.96 to 2.08 nm for O‐PBI, corresponding to a change of 0.88 nm. By contrast, FE‐PBI shows only a limited decrease from 2.29 to 2.13 nm, corresponding to a change of 0.16 nm. These results indicate that O‐PBI undergoes much more pronounced rearrangement of the transport‐relevant nanodomain/channel structure under alkaline conditions. In contrast, FE‐PBI preserves a more stable transport‐relevant nanostructural organization.

The NMR studies systematically investigated the ion states within the membrane ion channels. (^23^Na NMR was applied due to its higher sensitivity compared with ^39^K) [15]. ^23^Na NMR can show the ion‐dipole interaction between Na^+^ ions and model polymers in an aqueous solution [17]. ^23^Na magic‐angle‐spinning solid‐state NMR (^23^Na ssNMR) shows two separate Na^+^ signals and substantially broadened resonance for O‐PBI membranes (Figure 4e), with the downfield signal representing free Na^+^ (located at the same position as that in 100 mm aqueous NaOH solution) and the upfield signal corresponding to Na^+^ within the ion transport channels of the membrane [17]. By contrast, only the downfield signal and the narrow resonance suggested that free Na^+^ is detected for the FE‐PBI membrane (Figure 4e), which indicates less cation interaction and a faster ionic channel for FE‐PBI. This is further validated by the ionic conductivity of the membrane before/after alkaline treatment. As shown in Figure 4f and Figure S22, the FE‐PBI membrane maintains a high ionic conductivity before (12.5 mS cm^−1^) and after alkaline treatment (11.2 mS cm^−1^), while the ionic conductivity of the O‐PBI membrane decreases from 7.2 to 2.9 mS cm^−1^. The ionic permeation rate of O‐PBI and FE‐PBI membranes before/after alkaline treatment also exhibits the same trend (Figure 4g). Therefore, the aforementioned results collectively demonstrate the excellent alkaline stability of the FE‐PBI membrane with fixed ethylene linkages, enabling it to maintain high ionic conductivity. The deformation in the O‐PBI polymer and the decrease in ionic conductivity are responsible for increased electrochemical polarization and shortened life (Figure 3).

### 2.4 Molecular Dynamics Insight into Ion Transport Channels in Alkali Media

**FIGURE 5:**
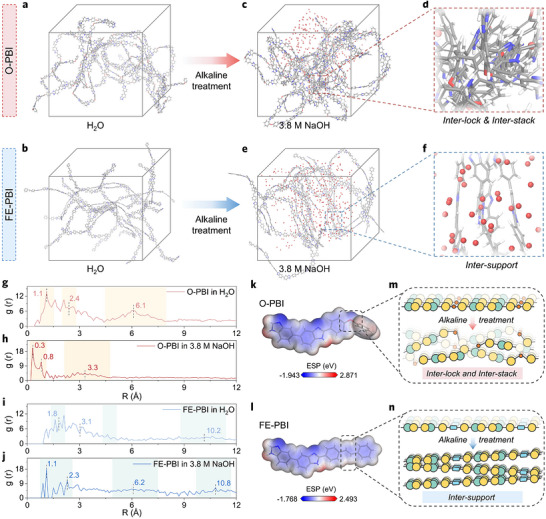
Molecular dynamics simulation of membranes in alkaline media. (a,b) 3D view of the chain structure of O‐PBI and FE‐PBI membranes in H_2_O media. (c,f) 3D view of the chain structure of O‐PBI and FE‐PBI membranes after aging in alkaline media (3.8 m NaOH) with the corresponding (d,f) detailed images. (g–j) The radial distribution function g(r) for the center‐of‐mass of imidazole‐imidazole in O‐PBI and FE‐PBI polymer chains based on H_2_O media and after aging in alkaline media (3.8 m NaOH). (k,l) ESP distribution map with iso‐surface of electronic density for O‐PBI and FE‐PBI membranes. (m,n) Schematic illustration of the structure change of O‐PBI and FE‐PBI membranes before/after alkaline treatment.

The alkaline stability stems from the physicochemical characteristics at the molecular and polymer chain levels. We conducted molecular dynamics (MD) simulations to examine the evolution of polymer chain structures. Figure 5a,b shows the optimal structural states of the O‐PBI and FE‐PBI polymer in an aqueous (H_2_O) medium. As expected, O‐PBI polymer chains (Figure 5a) appear more disordered compared with the uniform structure of FE‐PBI membrane (Figure 5b). When polymer membranes are exposed to an alkaline environment (3.8 m NaOH), the O‐PBI‐based polymer chains show significant interlocking and inter‐stacking (Figure 5c,d) with a disordered ion transport channel. This is well in accord with the finding in the last section for the reduced ionic conductivity (58%) and permeability. For the FE‐PBI membrane, the fixed ethylene (─C═C─) linkages‐based polymer structure can sustain a favorable state in alkaline media (Figure 5e). The inter‐support between polymer chains helps preserve well‐ordered ion transport channels (Figure 5f), thereby maintaining the superior ionic conductivity during cycling of FBs. Moreover, radial distribution function g(r) for inter‐imidazole under aqueous and alkaline media gives a more direct indication about the ion transport channel, flexible ether (─O─) linkages‐based polymer exhibit a close range between imidazole units from 1.1, 2.4, and 6.1 Å to 0.3, 0.8 and 3.3 Å (Figure 5g,h), forming relatively narrow and disordered channels. In contrast, FE‐PBI membranes resulted in a more uniform distribution of chain domains between 1.1 and 6.2 Å (Figure 5i,j), which can retain high‐flux ion transport channels.

The metal ion interaction is a primary culprit that leads to chain interlocking, which can be evidenced by the electrostatic potential (ESP) distribution maps of the polymer unit, where the regions with more negative ESP tend to interact with metal ions [12, 13]. As shown in Figure 5k, ether (─O─) linkages in O‐PBI membranes exhibit a strong negative charge, while the rigid ethylene (─C═C─) linkages in FE‐PBI membranes are nearly uncharged (Figure 5l), indicating that ether (─O─) linkages are prone to interact with metal cations.

Collectively, through the analysis of the chain evolution and molecular level characteristics, we found that the flexible properties of ether (─O─) linkages render the chain structure of O‐PBI membranes easily deformed and interacting with metal ions under alkaline conditions, resulting in interlocking and inter‐stacking between polymer chains (Figure 5m). For the FE‐PBI membrane (Figure 5n), benefiting from its fixed rigid structure and weak metal ion interactions, the FE‐PBI polymer chains could inter‐support each other to maintain well‐defined ion transport channels in alkaline media. The differences in metal ion interaction and ion transport channel evolution in an alkaline environment are the root cause of the different alkaline stability and ionic conductivity persistence.

### 2.5 High‐Power and Long‐Life Alkaline Zn─Fe FBs

**FIGURE 6:**
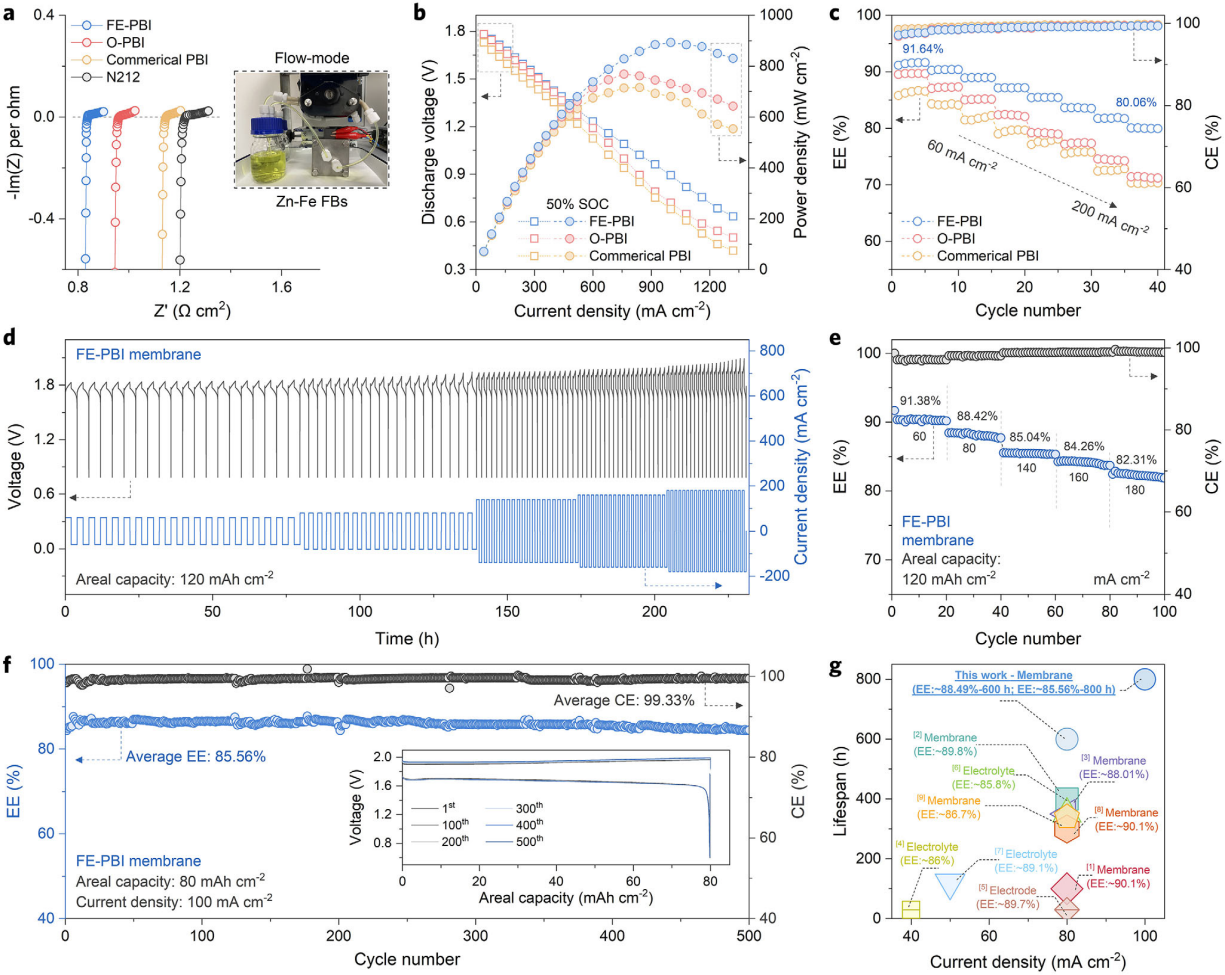
Electrochemical performance of FE‐PBI membrane in alkaline Zn─Fe FBs. (a) EIS spectra of alkaline Zn─Fe FBs cells assembled with N212, commercial PBI, O‐PBI, and FE‐PBI membranes at 50% SOC. (b) Voltage and power density vs. current density of alkaline Zn─Fe FBs cells assembled with commercial PBI, O‐PBI, and FE‐PBI membranes at 50% SOC. (c) Galvanostatic cycling performance of alkaline Zn─Fe FBs cells assembled with commercial PBI, O‐PBI, and FE‐PBI membranes at 40 mAh cm^−2^ with the current range of 60–240 mA cm^−2^. (d) Cycling and rate performance of alkaline Zn─Fe FBs cells assembled with FE‐PBI membrane at the high areal capacity of 120 mAh cm^−2^ with the current range of 60 – 180 mA cm^−2^. (e) The corresponding CE and EE performance by panel (d). (f) Cycling performance of alkaline Zn─Fe FBs cells assembled with FE‐PBI membrane at an areal capacity of 80 mAh cm^−2^ with a high current density of 100 mA cm^−2^. The inset shows the corresponding cell voltage profiles at the 1st, 100th, 200th, 300th, 400th, and 500th cycles by panel (f). (g) Comparison of alkaline Zn─Fe FBs with different developed strategies regarding current density and lifespan.

The superior alkaline tolerance and persistent ionic conductivity (FE‐PBI) are expected to contribute to improved electrochemical performance, including output peak power density, rate performance, and lifespan in alkaline Zn─Fe FBs. Firstly, a much lower internal resistance of 0.83 Ω cm^2^ was found for Zn─Fe FBs with FE‐PBI membrane, as shown by high‐frequency EIS in cells operating at 50% state‐of‐charge (SOC) (Figure 6a), compared with that of O‐PBI, commercial PBI, and N212 membranes in otherwise identical cells. Correspondingly, the polarization curve of FBs with FE‐PBI membranes at 50% SOC exhibits superior performance compared to O‐PBI and commercial PBI membranes (Figure 6b). Specifically, the developed Zn─Fe FBs achieve an output peak power density of 893, 1043, and 1202.4 mW cm^−2^ at SOC of 50%, 70%, and 90%, respectively (Figure S23). High working current densities, from 60 to 200 mA cm^−2^ under an areal capacity of 40 mAh cm^−2^ with exceptionally high coulombic efficiency (CE) and energy efficiency (EE) (Figure 6c; Figure S24) are realized. In particular, the CE and EE of Zn─Fe FBs can maintain the high value of over 99.34% and 80.06% even as the current density increases to 200 mA cm^−2^, indicating the limited permeability of active materials and high ionic conductivity of this FE‐PBI membrane.

For cycling performance, the as‐developed Zn─Fe FBs deliver a stable charge‐discharge operation over 600 cycles at 80 mA cm^−2^ and 40 mAh cm^−2^ with stable CE (average > 99.42%), realizing the EE of 88.49% (Figures S25 and S26).  The above results indicate that the FE‐PBI membrane with a rigid structure can endow long‐lasting, high‐flux ion transfer in the alkaline working environment. Furthermore, a high areal capacity of 120 mAh cm^−2^ was adopted to testify to the potential of the as‐fabricated Zn─Fe FBs. During 100 cycles of operation (Figure 6d,e) at high areal capacity of 120 mAh cm^−2^, stable CE and EE can be observed under various working currents for Zn─Fe FBs using FE‐PBI membranes with high EE of 91.38%, 88.42%, 85.04%, 84.26% and 82.31% at 60, 80, 140, 160, 180 mA cm^−2^, respectively. Meanwhile, we evaluated the practical applicability of Zn─Fe FBs by assessing the cycling performance under conditions of high current density (100 mA cm^−2^) and high areal capacity (80 mAh cm^−2^). The scale‐up FE‐PBI membranes‐based Zn─Fe FBs flow system can operate stably for 500 cycles (>800 h) with no obvious CE (average 99.33%) and EE (average 85.56%) decay (Figure 6f). In stark contrast, commercial PBI and the O‐PBI membrane‐based Zn─Fe FBs under the same working conditions demonstrate lower EE (commercial PBI‐based FBs: 83.21% at initial process; O‐PBI‐based FBs: 85.21% at initial process) and short cycle lifespan (commercial PBI‐based FBs: 120 cycles; O‐PBI‐based FBs: 240 cycles), respectively (Figures S27 and S28). The limited variation of the elemental contents before and after cycling by inductively coupled plasma optical emission spectrometer (ICP‐OES) further confirms the superior structural stability of the FE‐PBI membrane during battery operation (Figures S29 and S30).

The above exhibited a superior rate and cycling performance of FE‐PBI membrane‐based Zn─Fe FBs, which certified the effectiveness of this stable, rigid polymer structure in improving anti‐alkaline ability and retaining high ionic conductivity. Considering more practical conditions together, the FE‐PBI membranes‐based Zn─Fe FBs also demonstrate notable improvements compared with previously reported works in terms of current density and life duration, as shown in Figure 6g and Table S2. Notably, the remarkable long‐term durability performance of this membrane holds great promise for large‐scale energy storage applications.

## 3 Discussion

We designated a fixed ethylene linkage‐based polybenzimidazole (FE‐PBI) membrane with a rigid and stable polymer framework to overcome the challenges of ionic conductivity and the intrinsic instability of the current PBI‐based membranes. Benefiting from the inter‐support provided by the fixed ethylene backbone between FE‐PBI polymer chains and less metal cation interaction, FE‐PBI membranes can maintain the highly ordered and high‐flux ion transport channels after alkaline treatment with a retained ionic conductivity of 11.2 mS cm^−1^, which is 3.8‐fold higher than that of O‐PBI (2.9 mS cm^−1^). As a result, alkaline Zn‐based asymmetric flow batteries (FBs) showed excellent rate and cycling performance due to the high reversible Zn metal plating/stripping process. The alkaline zinc–iron (Zn─Fe) FBs utilizing the FE‐PBI membrane delivered stable cycling at a high areal capacity of 120 mAh cm^−2^ across a different current density range of 60 to 180 mA cm^−2^. The FE‐PBI membrane enabled long‐term stable operation for alkaline Zn─Fe FBs, achieving a high‐level energy efficiency (average ∼85.56%) over 800 h at 100 mA cm^−2^. This work can provide an innovative membrane design strategy for long‐term energy storage applications, combining high energy efficiency and long life.

## 4 Methods

All detailed methods are described in the Supporting Information.

## Conflicts of Interest

The authors declare no conflicts of interest.

## Supporting information




**Supporting File**: advs75334‐sup‐0001‐SuppMat.docx.

## Data Availability

The authors have nothing to report.
