# Supplementary material for: Fixed‐Linkage Enabled Ultra‐Stable Ion Transport Channels in Membranes for Long‐Life Alkaline Flow Batteries
Source: Adv Sci (Weinh). 2026 Apr 14;13(33):e24374. doi: 10.1002/advs.202524374 (PMC13271648; doi:10.1002/advs.202524374)
Supplement: Supplementary file 1 — Supporting File: advs75334‐sup‐0001‐SuppMat.docx. [file ADVS-13-e24374-s001.docx]

Supporting Information

Fixed-linkage Enabled Ultra-stable Ion Transport Channels in Membranes for Long-life Alkaline Flow Batteries

Zhiquan Wei^1#^, Jiaxiong Zhu^1#^, Yiqiao Wang^1^, Xinru Yang^1^, Dedi Li^1^, Hong Hu^1^, Qingshun Nian^1^, Shaoce Zhang^1^, Shixun Wang^1^, Zhuoxi Wu^1^, Yue Hou^1,2^, Shengnan Wang^1^, Ze Chen^3^, Qing Li^4^*, Chunyi Zhi^1,5,6,7^*

1 Department of Materials Science and Engineering, City University of Hong Kong, Hong Kong, 999077, China

2 Hong Kong Center for Cerebro-Cardiovascular Health Engineering (COCHE), Hong Kong, 999077, China

3 School of Interdisciplinary Studies, Lingnan University, Hong Kong, 999077, China

4 Institute of Applied Physics and Materials Engineering, University of Macau, Macau SAR, 999078, China

5 Materials Innovation Institute for Life Sciences and Energy (MILES), HKU-SIRI, Shenzhen, 518048, China

6 Center for Energy Storage, The University of Hong Kong, Pokfulam Road, Hong Kong, 999077, China

7 Department of Mechanical Engineering, The University of Hong Kong, Pokfulam Road, Hong Kong, 999077, China

^#^ These authors contributed equally

* Corresponding author:

Email: liqing@um.edu.mo (Q. Li); cy.zhi@cityu.edu.hk (C. Zhi)

**Table of contents**

**Page NO.**

[**Experimental Section** 1](#_Toc142315744)

[**Figure S1.** Schematic illustration of synthesis 7](#_Toc142315745)

[**Figure S2.** NMR spectrum of O-PBI and FE-PBI membranes 8](#_Toc142315745)

[**Figure S3.** TG thermograms of O-PBI and FE-PBI membranes 9](#_Toc142315746)

[**Figure S4.** UV-vis absorption spectrum of membranes 10](#_Toc142315746)

[**Figure S5.** Nyquist plots of different membranes in alkaline solutions 11](#_Toc142315747)

[**Figure S6.** Current-voltage profile of FE-PBI membrane in alkaline solutions 12](#_Toc142315748)

[**Figure S7.** Current-voltage profile of different membranes in alkaline solutions 13](#_Toc142315749)

[**Figure S8.** Schematic illustration of ionic permeation 14](#_Toc142315750)

[**Figure S9.** Standard UV-visible spectra of aqueous K_3_[Fe(CN)_6_] electrolytes 15](#_Toc142315751)

[**Figure S10.** [Fe(CN)_6_]^3−^ permeate solutions through N212 membrane 16](#_Toc142315752)

[**Figure S11.** [Fe(CN)_6_]^3−^ permeate solutions through commercial PBI membrane 17](#_Toc142315753)

[**Figure S12.** [Fe(CN)_6_]^3−^ permeate solutions through O-PBI membrane 18](#_Toc142315754)

[**Figure S13.** [Fe(CN)_6_]^3−^ permeate solutions through FE-PBI membrane 19](#_Toc142315755)

[**Figure S14.** Quantitative comparison of permeation across different membranes 20](#_Toc142315755)

[**Figure S15.** IEC of membranes before and after alkaline treatments 20](#_Toc142315755)

[**Figure S16.** Stress-strain curves of different membranes after alkaline treatment 21](#_Toc142315756)

[**Table S1.** The key parameters for ranking of properties of different membranes 22](#_Toc142315776)

[**Figure S17.** Polarization comparison of Zn-based asymmetrical FBs with different membranes 23](#_Toc142315757)

[**Figure S18.** SEM images after Zn charging under commercial PBI membrane 24](#_Toc142315757)

[**Figure S19.** XRD patterns of Zn-based asymmetrical FBs after Zn plating with different membranes 25](#_Toc142315758)

[**Figure S20.** Two-dimensional scattering pattern of O-PBI membranes before/after alkaline treatment 26](#_Toc142315759)

[**Figure S21.** Two-dimensional scattering pattern of FE-PBI membranes before/after alkaline treatment 27](#_Toc142315760)

[**Figure S22.** Nyquist plots of different membranes before/after alkaline treatment 28](#_Toc142315761)

[**Figure S23.** Power density of alkaline Zn-Fe FBs cells assembled with FE-PBI membranes at 70% and 90% SOC 29](#_Toc142315761)

[**Figure S24.** GCD-rate profiles of alkaline Zn-Fe FBs with different membranes 30](#_Toc142315763)

[**Figure S25.** Cycling performance of alkaline Zn-Fe FBs with FE-PBI membrane at 80 mA cm^−2^ 31](#_Toc142315764)

[**Figure S26.** Self-discharge behavior of Zn-Fe FBs with different membranes during resting 32](#_Toc142315764)

[**Figure S27.** Cycling performance of alkaline Zn-Fe FBs with commercial membrane at 100 mA cm^−2^ 33](#_Toc142315765)

[**Figure S28.** Cycling performance of alkaline Zn-Fe FBs with O-PBI membrane at 100 mA cm^−2^ 34](#_Toc142315766)

[**Figure S29.** P element content in different membranes before/after cycles measured by ICP-OES 35](#_Toc142315766)

[**Figure S30.** K^+^ and Na^+^ content in different membranes after cycles measured by ICP-OES 36](#_Toc142315766)

[**Table S2.** Performance comparison between this work and previously reported work 37](#_Toc142315776)

[**Reference** 38](#_Toc142315797)

# Experimental Section

1. **Materials**

All chemicals were used as received. 4,4'-Stilbenedicarboxylic acid (96%) was received from Thermo Scientific Chemicals. 3,3'-Diaminobenzidine (C_12_H_14_N_4_, 99%), 4,4'-Diaminodiphenyl ether (C_12_H_12_N_2_O, 98%), Phosphorus pentoxide (P_2_O_5_, 99.99%, metals basis), and Methanesulfonic acid (CH_4_O_3_S, MSA) were received from Aladdin Scientific Corp. Phosphoric acid (H_3_PO_4_, ≥85wt.% in H_2_O), N, N-dimethylacetamide (C_4_H_9_NO, DMAc, AR, 99%), Polyphosphoric acid (reagent grade, 115% H_3_PO_4_ basis), Zinc oxide (ZnO, 99.9%, metals basis, 30±10 nm), Sodium hydroxide (NaOH, AR, 96%), Sodium ferrocyanide decahydrate (Na_4_Fe(CN)_6_·10H_2_O, ≥99%), Potassium hydroxide (KOH, ≥95%), Potassium ferricyanide (K_3_FeC_6_N_6_, AR, ≥99.5%), Potassium chloride (KCl, AR, 99.5%), Sodium chloride (NaCl, AR, 99.5%), Lithium chloride (LiCl, AR, 99.0%), Calcium chloride dihydrate (CaCl_2_·2H_2_O, ACS, ≥99%), and Magnesium chloride hexahydrate (MgCl_2_·6H_2_O, 99.99%) were received from Sigma-Aldrich. Carbon felt (CF, 4.6 mm, carbon ≥99 %, bulk density 0.12-0.14 g cm^-2^) was received from Yi Deshang Carbon Technology. Polyolefin-based porous membrane was received from Daramic®. Zn foil (200 μm, 99.99%) was purchased from Chenshuo Metal Company.

1. **Synthesis of poly[2,2′-(p-oxydiphenylene)-5,5′-bibenzimidazole] (O-PBI polymers)**

O-PBI was synthesized by condensation polymerization of 4,4'-Diaminodiphenyl ether and 3,3'-Diaminobenzidine at 140 °C in P_2_O_5_/MSA in a weight ratio of 1:10 for 60 min with a total monomer concentration of 10 wt.%. The reaction mixture became more viscous during the polymerization and developed a dark brown color. After the reaction was completed, the reaction mixture was poured into a 5 wt.% Na_2_CO_3_ solution. The polymer was thoroughly rinsed with deionized water (DI H_2_O), filtered, and dried.

1. **Synthesis of poly[2,2′-(p-vinyl diphenylene)-5,5′-bibenzimidazole] (FE-PBI polymers)**

FE-PBI was synthesized by condensation polymerization of 4,4'-Stilbenedicarboxylic acid and 3,3'-Diaminobenzidine by a programmable temperature (120 °C for 1 h; 140 °C for 24 h; 180 °C for 1 h; 220 °C for 24 h) in 115% H_3_PO_4_ media with a total monomer concentration of 3 wt.%. The reaction mixture became more viscous during the polymerization and developed a dark brown color. After the reaction, the mixture was poured into a 5 wt.% Na_2_CO_3_ solution. The polymer was thoroughly rinsed with DI H_2_O, filtered, and dried.

1. **Membrane fabrication**

The O-PBI and FE-PBI membranes were fabricated using the solution cast method, where the dried PBI and B-PBI polymers were dissolved in DMAc to form a 7 wt.% solution, respectively. The solution was cast onto a clean and dry glass plate and dried at 50 °C for 48 h. The thickness of the prepared O-PBI and FE-PBI membranes was ∼35 μm, respectively. Both O-PBI and FE-PBI membranes were treated with H_3_PO_4_ for 24 h prior to use. H_3_PO_4_ soaking is commonly used as a conditioning step because it can protonate the benzimidazole sites, weaken the strong interchain hydrogen bonding and packing, and promote membrane wetting and structural relaxation, thereby making the membrane state more stable and reproducible before electrochemical testing.[^1^](#_ENREF_1)

1. **Ionic conductivity**

The ionic conductivity of the prepared membranes under different solutions (1 M NaOH and 1 M KOH) was tested by electrochemical impedance spectroscopy (EIS) using an electrochemical station (CHI 760E) according to the method as reported.[^2^](#_ENREF_2) membranes were stacked together and sandwiched between two round titanium plates. The effective diameter for each piece of membrane was 1.5 cm. The frequency of EIS was set from 1 Hz to 1000 kHz. The membranes were pretreated in the corresponding alkaline solutions for 24 h. The ionic conductivity of the membranes in different solutions was calculated by the following equation:

σ = L/R

Where σ (S cm^-1^) is the ionic conductivity of the membrane, L (cm) is the thickness of the membrane, and R (ohm cm^2^) is the area resistance of the membrane. Each result was an average value of three parallel experiments.

1. **Ion transference numbers through the membranes**

The ion transport numbers for membranes were investigated using CHI 760E. The V-I profile was recorded when the membrane was sandwiched between two cells soaking with different alkaline (NaOH and KOH) concentration gradients (0.11|0.33 M, 0.33|1 M, and 1|3 M, respectively). Two Ag/AgCl reference electrodes filled with saturated KCl solution and two salt bridges filled with saturated KCl solution were employed to eliminate the potential drop caused by the unequal potential drop at the electrode-solution interface and the liquid junction potential generated by two different electrolytes.[^3^](#_ENREF_3)^,^[^4^](#_ENREF_4) Thus, the open-cell voltage of the device (*V_0_*) is equal to the value of diffusion potential (*V_d_*) resulting from the alkaline concentration gradient, which can be calculated as the following equation:[^5^](#_ENREF_5)

$$\text{V}_{\text{o}}\text{ =}\text{ V}_{\text{d}}\text{ = }\frac{\text{RT}}{\text{F}}\left( \text{t}_{\text{K}^{\text{+}}\text{ or }\text{Na}^{\text{+ }}}\text{- }\text{t}_{\text{OH}^{\text{-}}} \right)\ln\left( \text{∆} \right)$$

*R*, *T*, *F*, $\text{t}_{\text{K}^{\text{+}}\text{ or }\text{Na}^{\text{+}}}$, $\text{t}_{\text{OH}^{\text{-}}}$, and $\text{∆}$ are the gas constant, temperature, Faraday constant, K^+^ or Na^+^ transference number, OH^−^ transference number and concentration gradient, respectively. Of note is that if the alkaline concentration gradient is high (e.g., 1|3 M), the mean activity coefficient of ions for the alkaline solution ($\text{∆}$) must be considered.

1. **Apparent ion-exchange capacity (𝐼𝐸𝐶_app_)**

𝐼𝐸𝐶_app_ was determined by a back-titration protocol commonly used for alkaline membranes. For the fresh samples, the membranes were first equilibrated in 1.0 M NaOH for 24 h at room temperature. The alkaline-aged samples were obtained after soaking in 3.8 M NaOH at 60 °C for 1 week. Before titration, all membranes were briefly rinsed three times with CO_2_-free deionized water to remove free alkali, and the surface liquid was carefully blotted off. The membranes were then immersed in 30 mL of 0.01 M HCl for 18 h at room temperature. The remaining HCl was back-titrated with 0.01 M NaOH using phenolphthalein as the indicator. After titration, the membranes were rinsed with 18.2 MΩ·cm water, dried in a vacuum desiccator for 24 h, and weighed. The apparent IEC was calculated according to:[^6^](#_ENREF_6)

$$\text{IEC}_{\text{app}}\text{ = }\frac{C_{HCl}V_{HCl}-C_{NaOH}V_{NaOH}}{m_{dry}}$$

where *C* and *V* denote the concentration and volume of HCl or NaOH, respectively, and *𝑚_dry_* is the dry mass of the membrane after titration.

1. **Ion permeability rate through the membranes**

The ion permeability rate was measured by a customized H-type diffusion cell with an effective area of a circle with a diameter of 1.5 cm. The left chamber of the diffusion cell was filled with 40 mL of prepared 1 M feeding solution (KCl, NaCl, CaCl_2_, and MgCl_2_), while the right chamber was filled with 40 mL of water. The ion concentration was measured by a Mettler Toledo pH meter and calculated using the following equation:[^7^](#_ENREF_7)

$$c = \kappa/{\Lambda_{m}}$$

where *𝜅* was the conductivity of the solution on the diffusion side, *c* was the ion concentration, and *Λ_m_* was the molar conductivity of metal chloride from references.[^8^](#_ENREF_8) The conductivity (*𝜅*) of the diffusion side at a certain time was first measured. Then, the concentration (*c*) on the diffusion side was calculated by *𝜅* and *Λ_m_*. The ion permeation rate (mol m^-2^ h^-1^) was calculated by the concentration (*c*) and the recorded time (*t*).

1. **Permeability of [Fe(CN)6]^3-^ through the membranes**

The permeability of [Fe(CN)_6_]^3−^ through the membrane was determined by a diffusion cell separated by a membrane. The left cell was filled with 0.4 M K_3_Fe(CN)_6_ in 3 M NaOH solution (volume: 18 mL), while the right one was filled with 3 M 3 M NaOH (volume: 18 mL). Solutions in both half cells were vigorously stirred to avoid concentration polarization. The effective area of the membrane was 1.766 cm^2^. Samples of a 3 mL solution from the right cell were collected at a regular time interval. Another 3 mL of fresh alkaline solution was added to the right cell to keep the solution volume stable. The [Fe(CN)_6_]^3−^ concentration of the samples was detected using a UV-vis spectrometer (Hitachi UH4150 UV-VIS-NIR Spectrophotometer). The permeability of [Fe(CN)_6_]^3−^ was calculated according to Fick’s diffusion law as the following equation:[^9^](#_ENREF_9)

$$\text{V}_{\text{o}}\frac{\text{d}\text{C}_{\text{B}}\text{(t)}}{\text{dt}}\text{ = A}\frac{\text{P}}{\text{L}}\text{(}\text{C}_{\text{A}}\text{-}\text{ }\text{C}_{\text{B}}\text{(t))}$$

where *V_B_* is the solution volume in the right reservoir, *C_B_(t)* is [Fe(CN)_6_]^3−^ concentration in the left cell as a function of time *t* (h), while *A* and *L* are the effective area (cm^2^) and thickness (cm) of the membrane, respectively. *P* is the permeability of [Fe(CN)_6_]^3−^, and *C_A_* is the concentration of [Fe(CN)_6_]^3−^ in the left cell.

1. **Alkaline stability of membranes**

To confirm the chemical stability in alkaline electrolytes, all membranes were treated in a 3.8 M NaOH solution at 60 °C for one month. Afterward, ionic conductivity, ion permeation rate, and the mechanical stability of treated membranes were measured and compared to the initial mechanical stability of membranes.

1. **Electrochemical characterization of Zn-based asymmetrical flow batteries**

The batteries were assembled with carbon felts (CF, 2 cm × 2 cm × 0.46 cm) used as anodes, Zn metal (2 cm × 2 cm × 200 μm) and CF (2 cm × 2 cm × 0.46 cm) used as cathode, proposed membranes and 30 mL of 0.2 M Zn(OH)_4_^2-^ in 3.8 M NaOH used as electrolyte to flow past the two electrodes. The test was examined by plating onto CF at different current densities and capacities, followed by stripping Zn from these substrates to a cut-off voltage (-0.6 V for rate and -0.5 V for long cycling test) by a battery testing system (LAND, CT2001A).

1. **Electrochemical characterization of alkaline zinc-iron flow batteries (Zn-Fe FBs)**

Posolytes composed of 0.4 M Na_4_Fe(CN)_6_ + 3 M KOH and negolytes composed of 0.2 M ZnO + 3.8 M NaOH were dissolved in DI H_2_O. The alkaline Zn-Fe FBs cells assembly configuration: briefly, stainless steel frames served as the flow channel to fix the position of the pretreated three-dimensional CF) electrodes with a geometric area of 4.0 cm^2^ (2 × 2 cm^2^) and a thickness of 3.0 mm. A peristaltic pump (Chuang Rui Precision Pump) was employed to power the circulation of the electrolyte flow through the electrodes to make a flow-mode cell.

The galvanostatic characterizations of the alkaline Zn-Fe FBs cells were conducted on a battery testing system (LAND, CT2001A). The current densities were set at 40 to 200 mA cm^−2^. The charging process was limited by the constant capacity (20 mAh cm^−2^ or 40 mAh cm^−2^), and the discharging process was limited by the cut-off voltage of 0.6 V. The theoretical capacity was calculated with the proselytes (the [Fe(CN)_6_]^3−^ part), which was the capacity-limiting side for the full cell.

1. **Characterizations**

The crystal structure was studied by X-ray diffraction (XRD, X’Pert Pro MPD, Philips, Holland) using Cu Kα as the radiation source under 40 kV and 40 mA. Morphologies were probed by scanning electron microscopy (SEM, FEI Quanta 450 FEG SEM). The mechanical stability of the membranes was characterized by a universal testing machine (Instron Series 3382 UTM System). The 1H NMR, recorded on BRUKER DRX400, was employed to confirm the chemical structure of the synthesized polymers by using DMSO‑d_6_ as solvent and tetramethylsilane (TMS) as internal standard. The top-surface chemical analysis of membranes was determined by the Fourier transform infrared spectrometer (FTIR, PerkinElmer Spectrometer) with a scan range from 800 to 4000 cm-1. The surface morphology of the membranes was obtained via an ultradeep surface morphology determination microscope (KEYENCE, VK-8510) and an OLYMPUS laser confocal scanning microscope. The thermal stability of membranes was measured using a TGA/DTA (Pyris Diamond TGA/DTA) system. Polymer samples for TGA analysis were heated from room temperature to 800 °C at a heating rate of 10 °C min^-1^ under an argon atmosphere. The elemental contents of membranes were determined by inductively coupled plasma optical emission spectrometry (ICP-OES, [instrument model, manufacturer]) after acid digestion of the dried membrane samples. Small-angle X-ray scattering (SAXS) data were collected using a Xeuss 2.0 instrument (Xenocs) with an incident X-ray wavelength of *λ* = 0.154 nm. SAXS/wide-angle X-ray scattering patterns were recorded in a *q* range of 0.1 nm^-1^ < *q* < 10 nm^-1^, where *q = (4πsin θ)/λ* is the length of the scattering vector, and *2θ* is the scattering angle. Based on Bragg’s law, *d = 2π/q*, where d is Na^+^ in the nanodomains. Both membrane samples were soaked in 1 M NaOH solution for SAXS tests.

1. **Density functional theory (DFT) calculation**

To study the ESP of the membranes, density functional theory (DFT) calculation was carried out using the Gaussian 16 package.[^10^](#_ENREF_10) The structures were firstly optimized at M06-2X hybrid functional with def2-SVP basis set, including the atom-pairwise dispersion correction (DFT-D3).[^11-14^](#_ENREF_11) Then, a single point energy calculation was performed at M06-2X/def2-TZVP level to obtain the wave function of each optimized structure. The ESP surface was analyzed by Multiwfn49 package on an isosurface of electronic density at 0.001 a.u. and drawn by VMD (visual molecular dynamics)50 package.[^15^](#_ENREF_15)^,^[^16^](#_ENREF_16)

1. **Molecular dynamics (MD) simulations**[**^17^**](#_ENREF_17)**^,^**[**^18^**](#_ENREF_18)

The Desmond module in the Schrodinger software (2021-3 version) was utilized for molecular dynamics simulation to construct the 3D structures of polymers. In brief, the 2D-sketcher function in the Schrodinger software was used to acquire 3D structures for the monomers and the structures for sodium hydroxide and water. The Disordered System Builder function in the Schrodinger software was utilized to establish the disordered system to sequentially established the systems for the following molecular dynamic stimulation: (1) 16 polymers (O-PBI) and 11000 H_2_O; (2) 16 polymers (FE-PBI) and 11000 H_2_O; (3) 16 polymers (O-PBI), 800 NaOH, and 11000 H_2_O; (4) 16 polymers (FE-PBI), 800 NaOH, and 11000 H_2_O. All polymers were composed of 4 units. The system was established by using the OPLS2005 force field and selecting the Amorphous-Steric Pack pattern for the initial status of the system. After the system was established, the molecular dynamics were performed using the MD Multistage Workflow function in Schrodinger. Briefly, we used the Material Relaxation function to relax the system and performed Simulated Annealing from 600 K to 298 K within 10 cycles, 20 ps each. Then, the molecular dynamics was performed for 20 ns at NPT, 300 K. After the molecular dynamics was finished, the radial distribution function (RDF) was utilized to analyze the results.


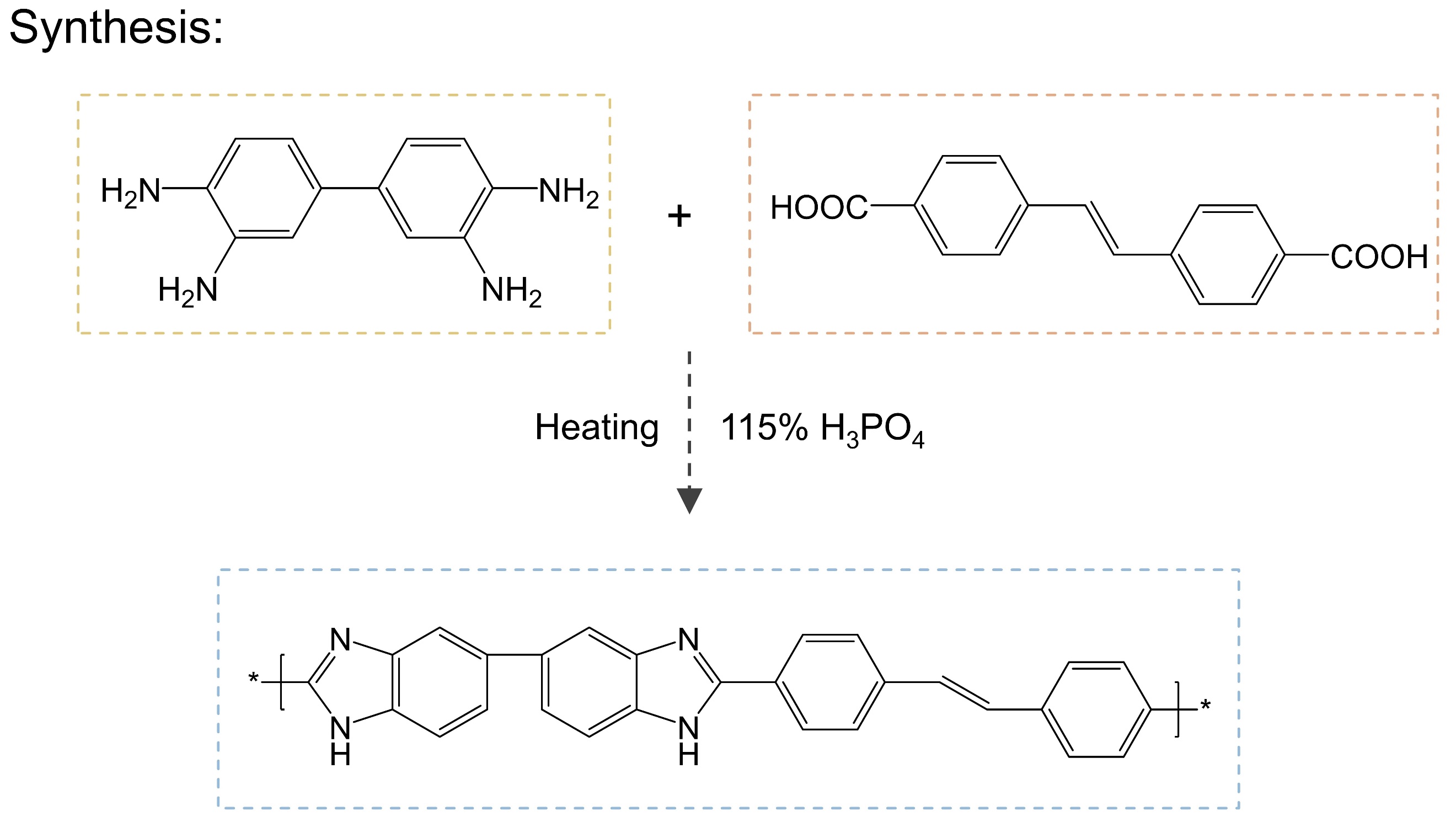


Figure S1. (a) Schematic diagram for the synthetic process of FE-PBI polymer.

**
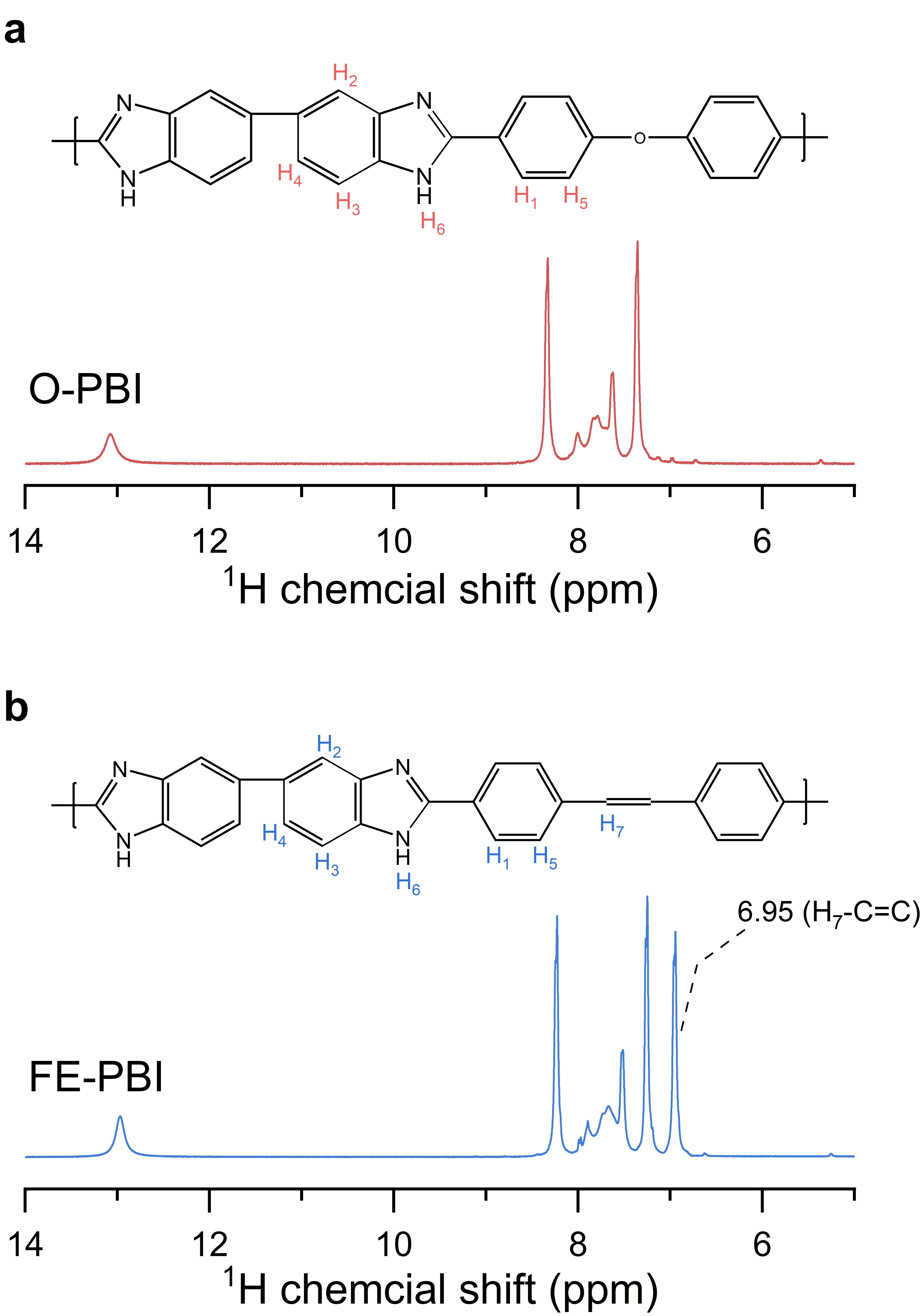
**

Figure S2. NMR spectrum of O-PBI and FE-PBI membranes.


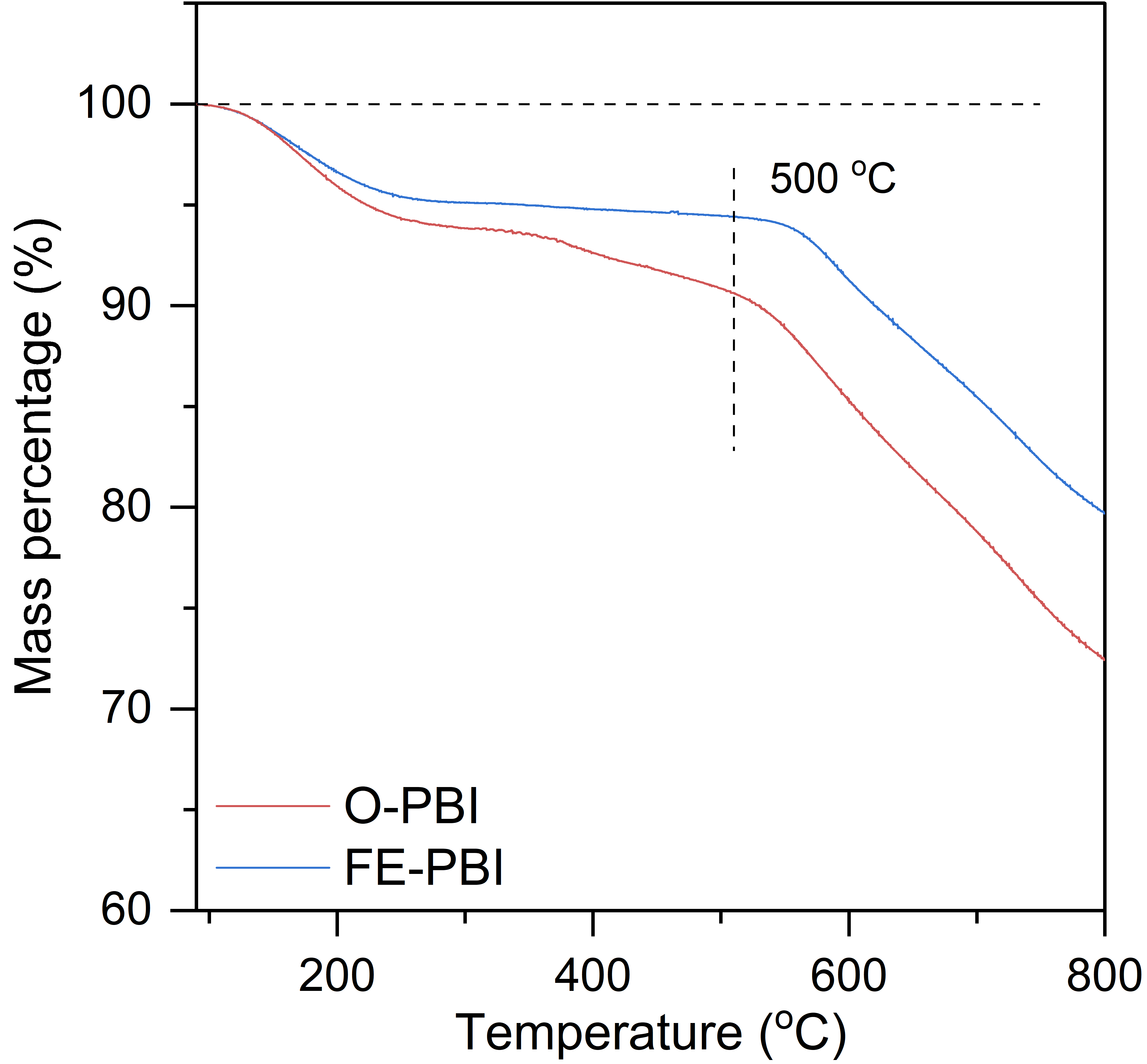


Figure S3. TG thermograms of O-PBI and FE-PBI membranes.


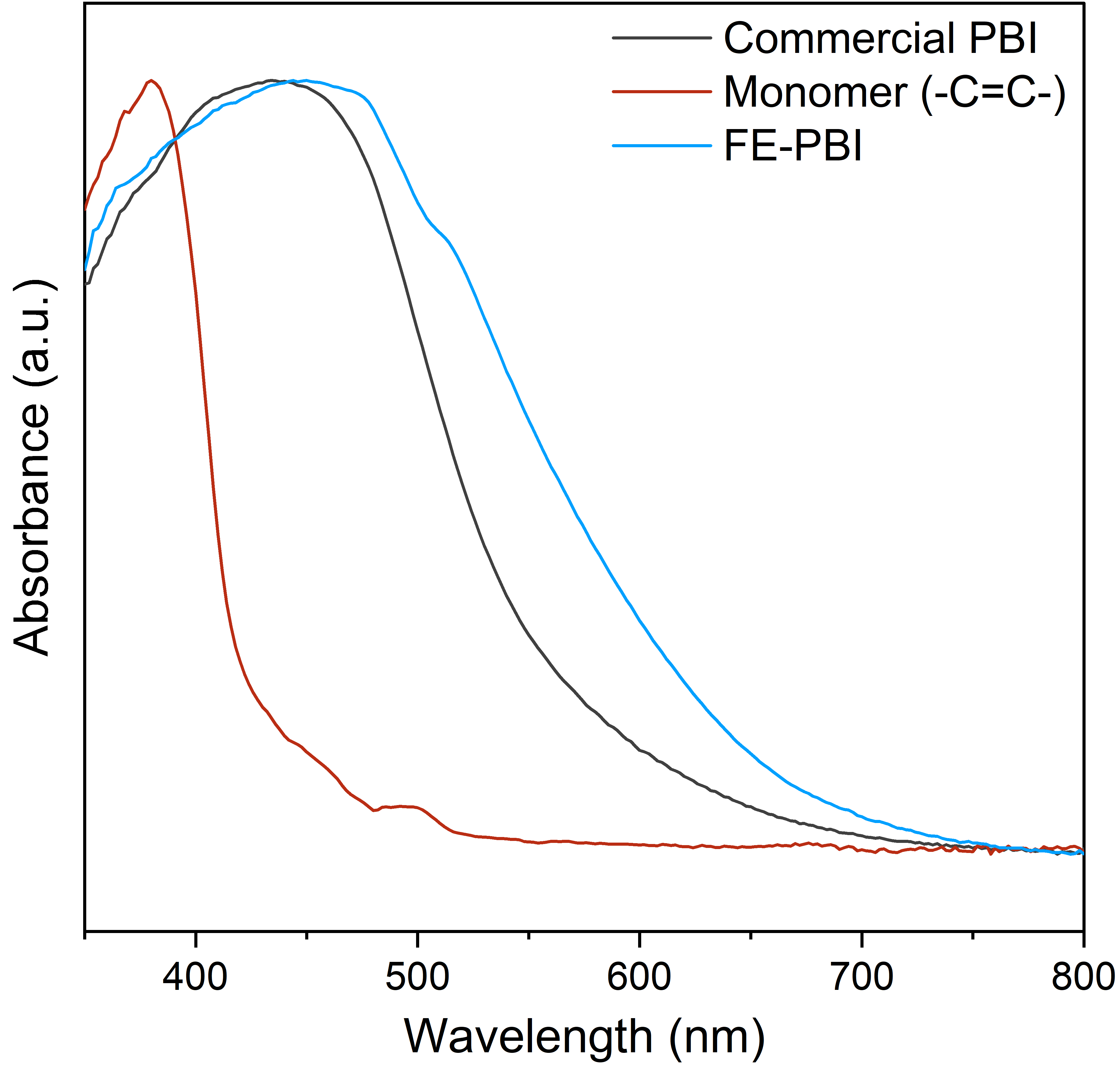


Figure S4. UV-visible absorption spectrum of commercial PBI membrane, the corresponding monomer, and FE-PBI membrane.

**
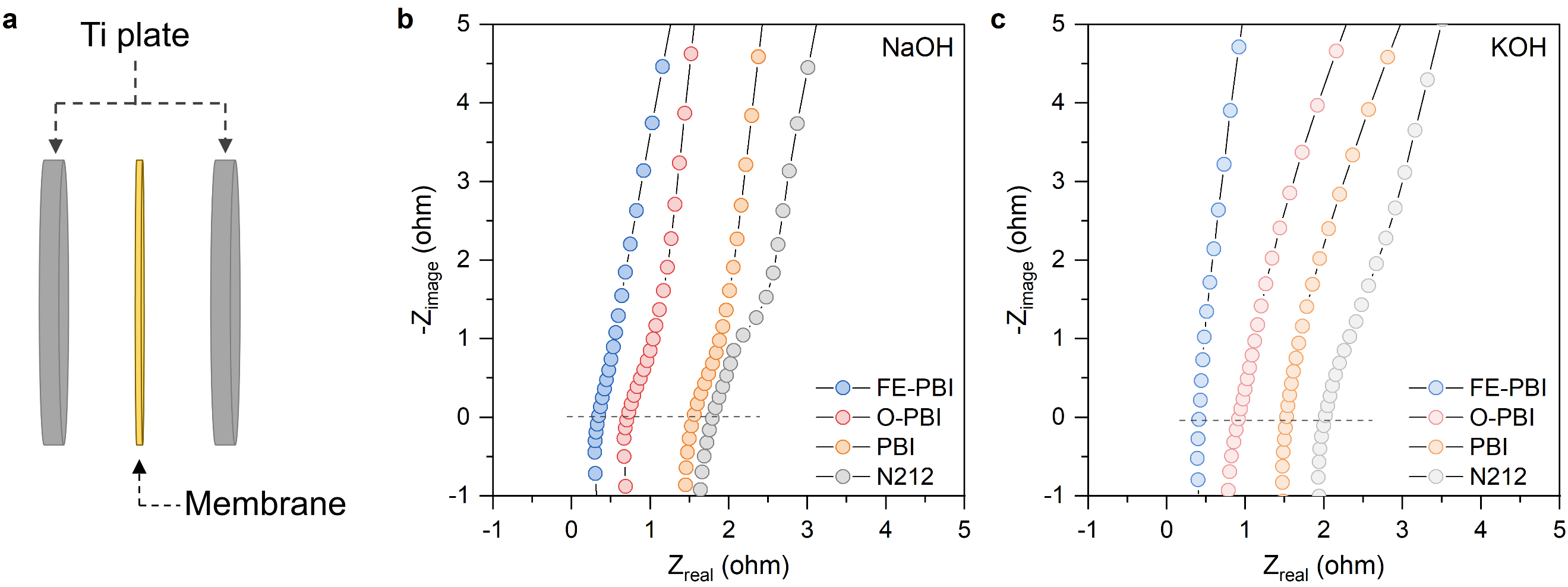
**

Figure S5. (a) Schematic diagram for membrane resistance measurement. (b) & (c) Nyquist plots if different membranes in 1 M NaOH and 1 M KOH.

**
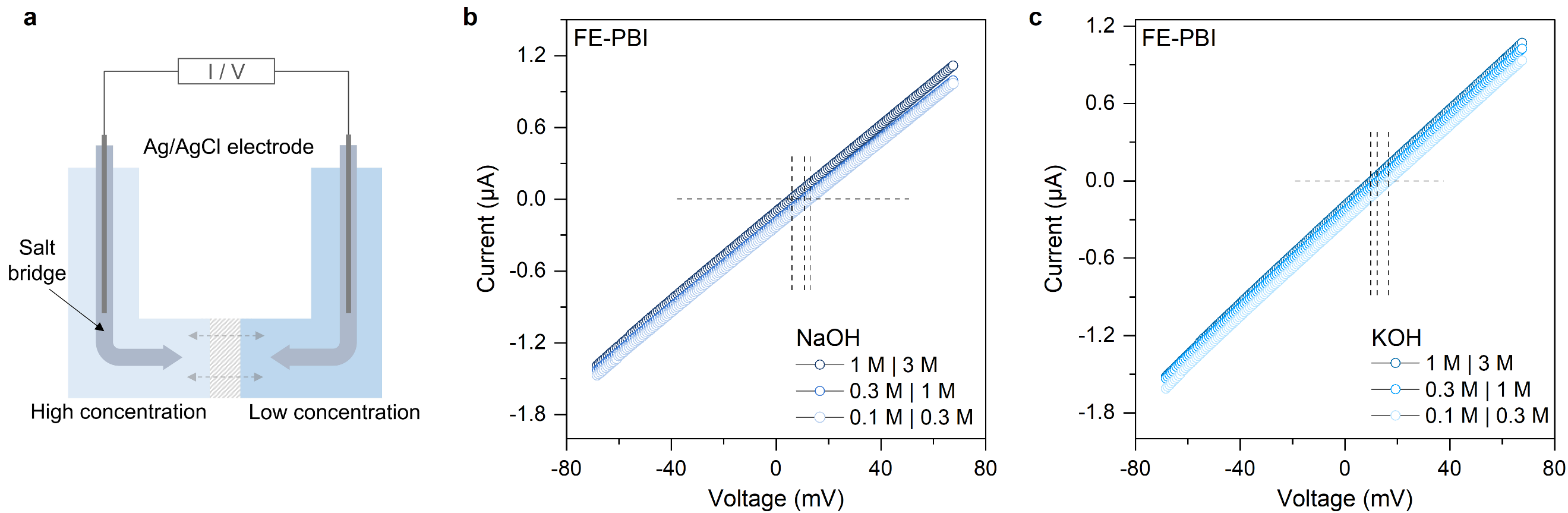
**

Figure S6. (a) Schematic illustration of V-I testing device; (b) & (c) The cation transference numbers and OH^-^ transference numbers calculated from V-I profiles for different NaOH or KOH concentration gradients in the two reservoirs connected by FE-PBI membranes.

**
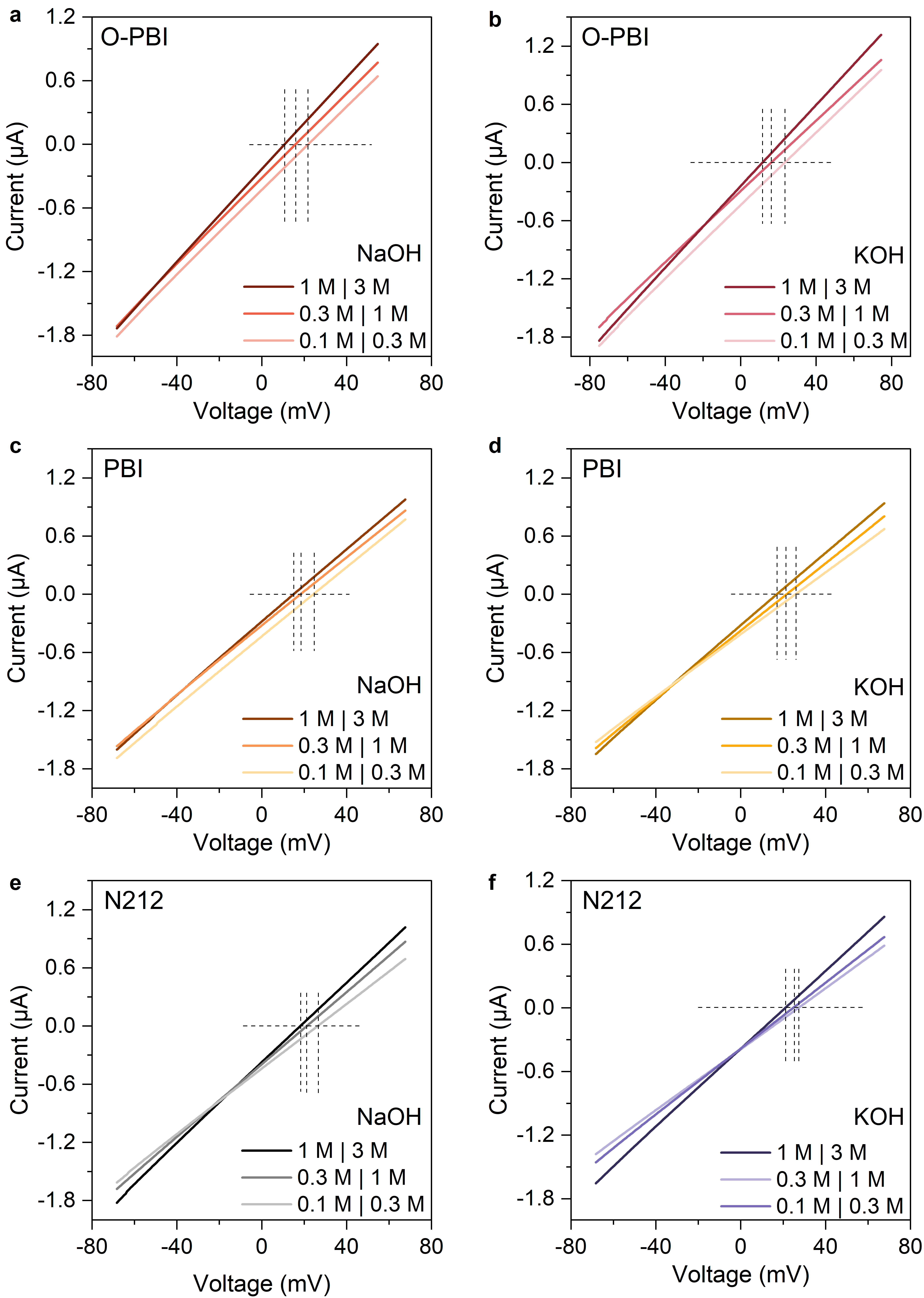
**

Figure S7. (a) & (b) The cation transference numbers and OH^−^ transference numbers calculated from V-I profiles for different NaOH or KOH concentration gradients in the two reservoirs connected by O-PBI membranes. (c) & (d) The cation transference numbers and OH^−^ transference numbers calculated from V-I profiles for different NaOH or KOH concentration gradients in the two reservoirs connected by PBI membranes. (e) & (f) The cation transference numbers and OH^-^ transference numbers calculated from V-I profiles for different NaOH or KOH concentration gradients in the two reservoirs connected by N212 membranes.

**
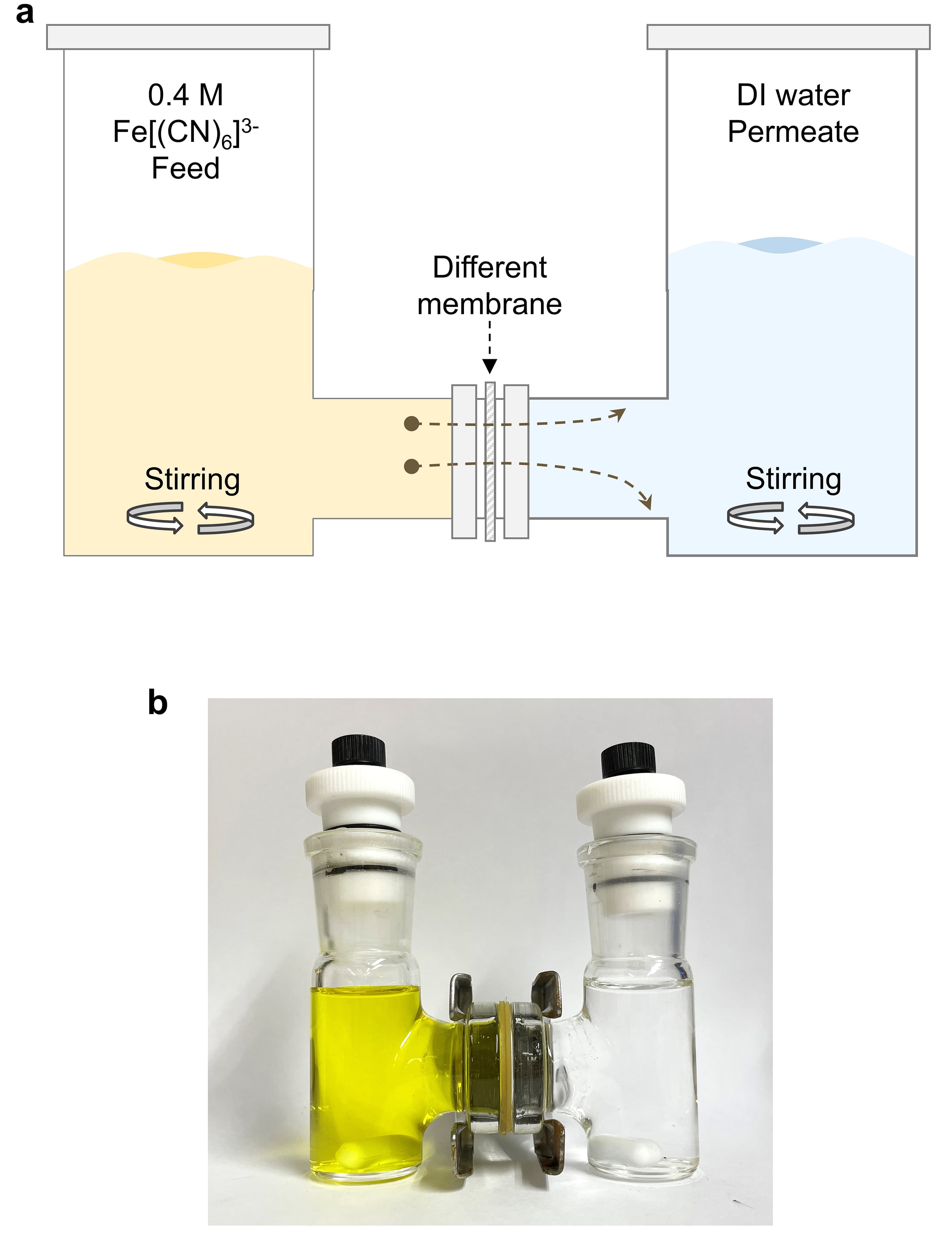
**

Figure S8. (a) Schematic illustration and (b) digital of two-compartment H-cell testing configuration of 0.4 M [Fe(CN_6_)]^3−^ permeation.


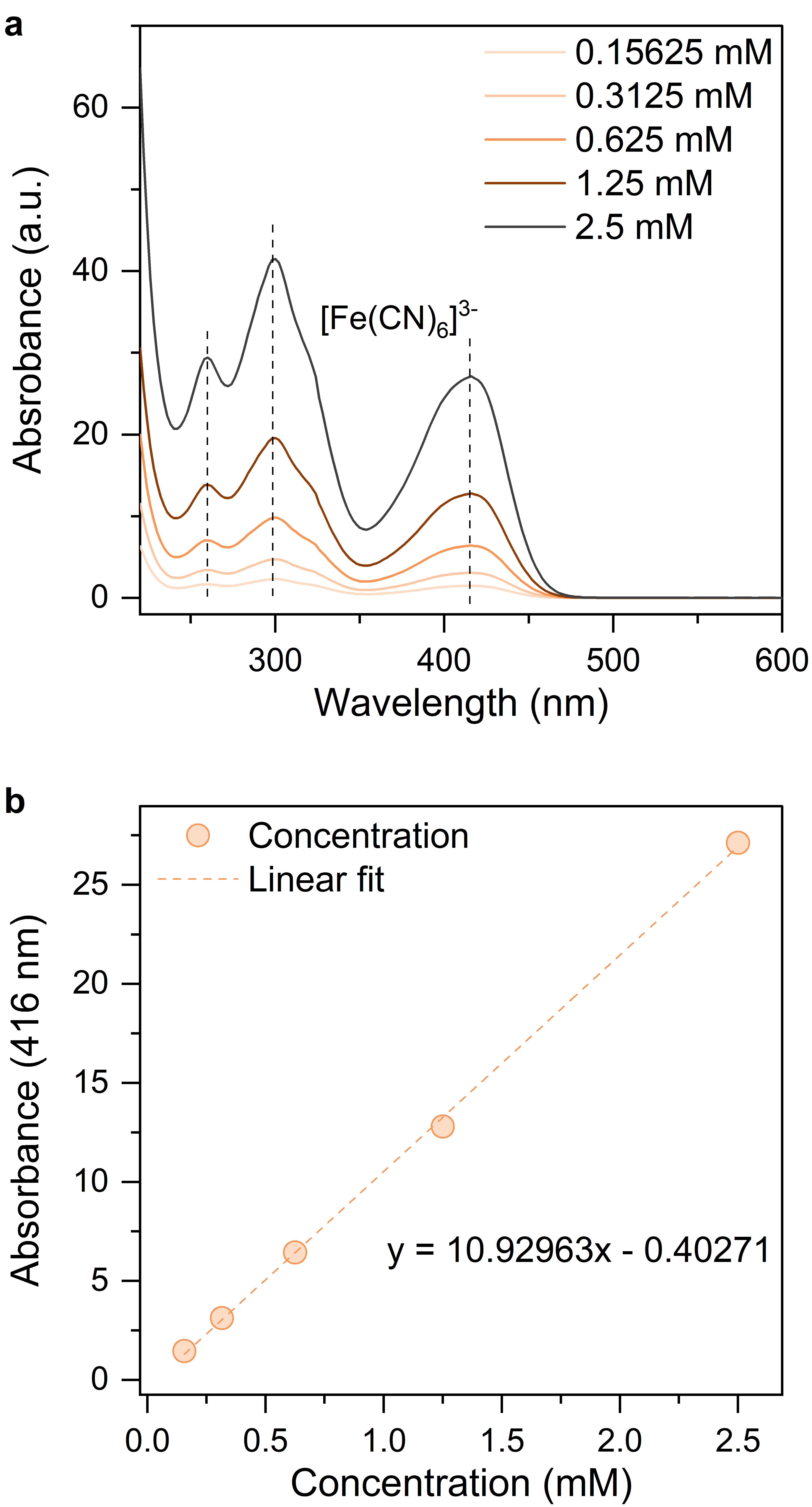


Figure S9. Standard UV-visible spectra of aqueous [Fe(CN_6_)]^3−^ electrolytes. (a) UV-visible spectra of [Fe(CN_6_)]^3−^ under multiple concentrations between 0.15625 mM to 2.5 mM; (b) Beer’s law plot for [Fe(CN_6_)]^3−^ multiple concentrations.


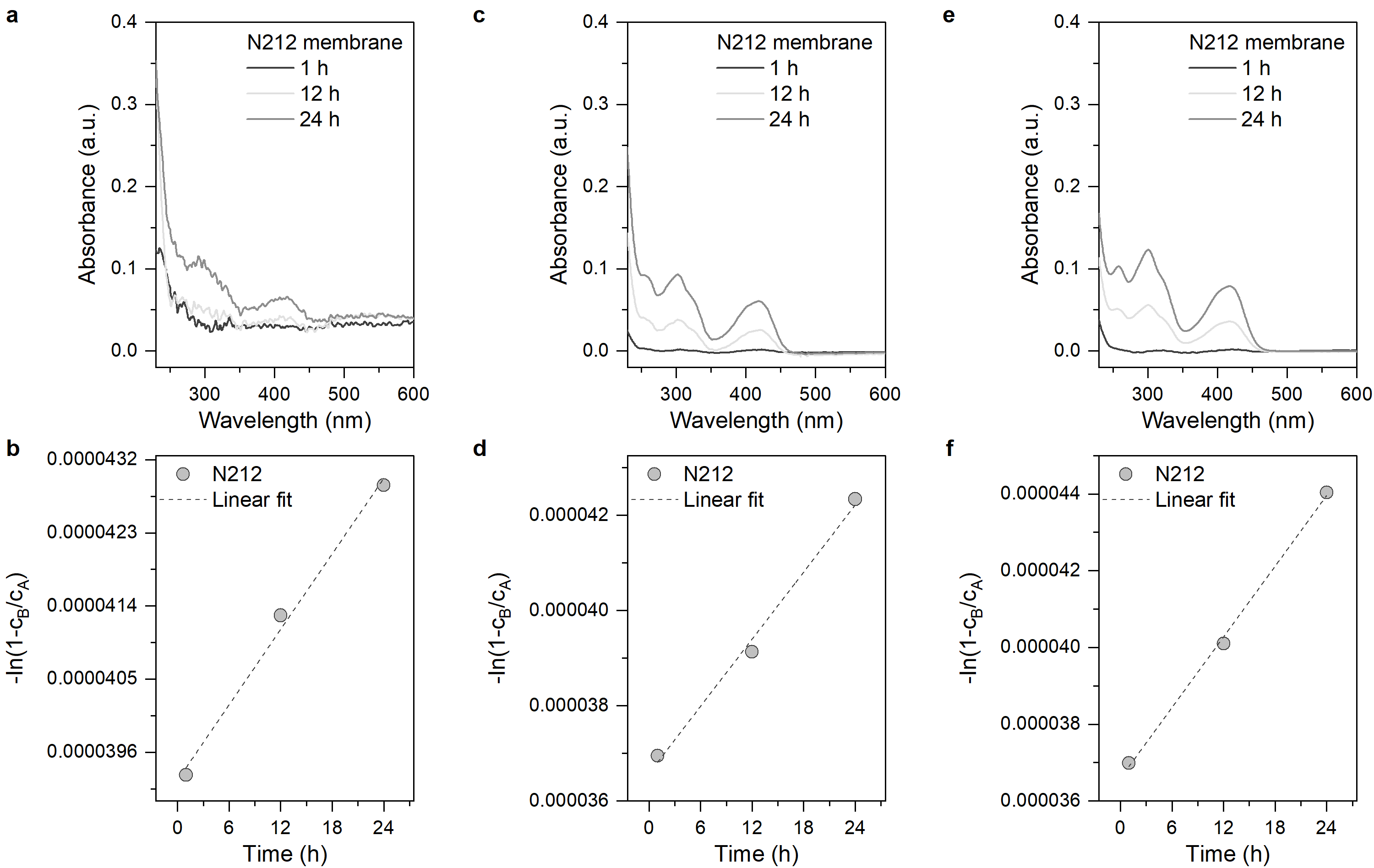


Figure S10. Three sets of the [Fe(CN_6_)]^3−^ permeation through the N212 membrane over time. (a, c, e) UV-vis absorption spectra of the permeate side. (b, d, f) -ln(1-c_B_/c_A_) *vs*. permeation time for the determination of permeability of [Fe(CN_6_)]^3−^ under the N212 membrane.


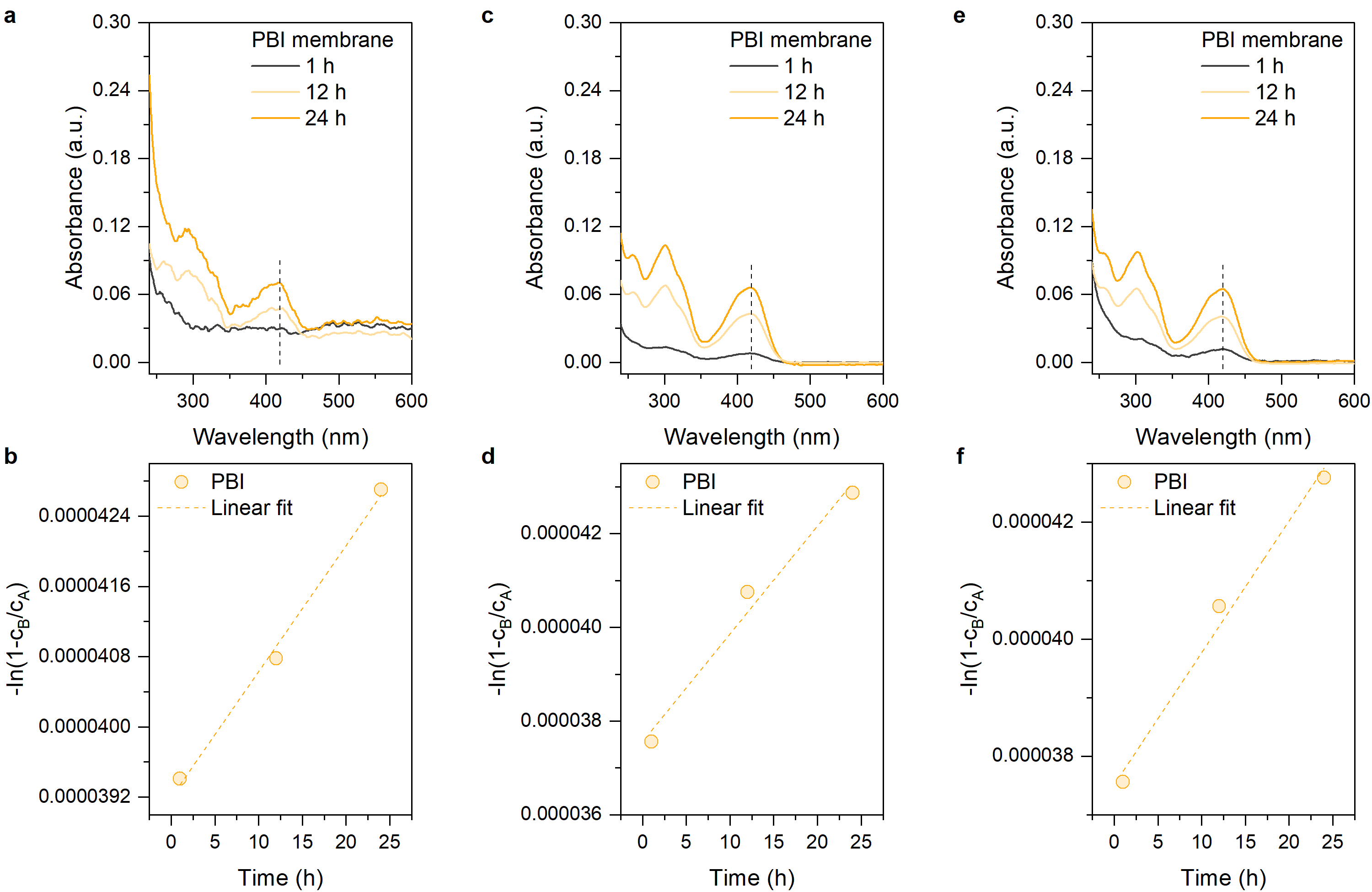


Figure S11. Three sets of the [Fe(CN_6_)]^3−^ permeation through commercial PBI membrane over time. (a, c, e) UV-vis absorption spectra of the permeate side. (b, d, f) -ln(1-c_B_/c_A_) *vs*. permeation time for the determination of permeability of [Fe(CN_6_)]^3−^ under the commercial PBI membrane.


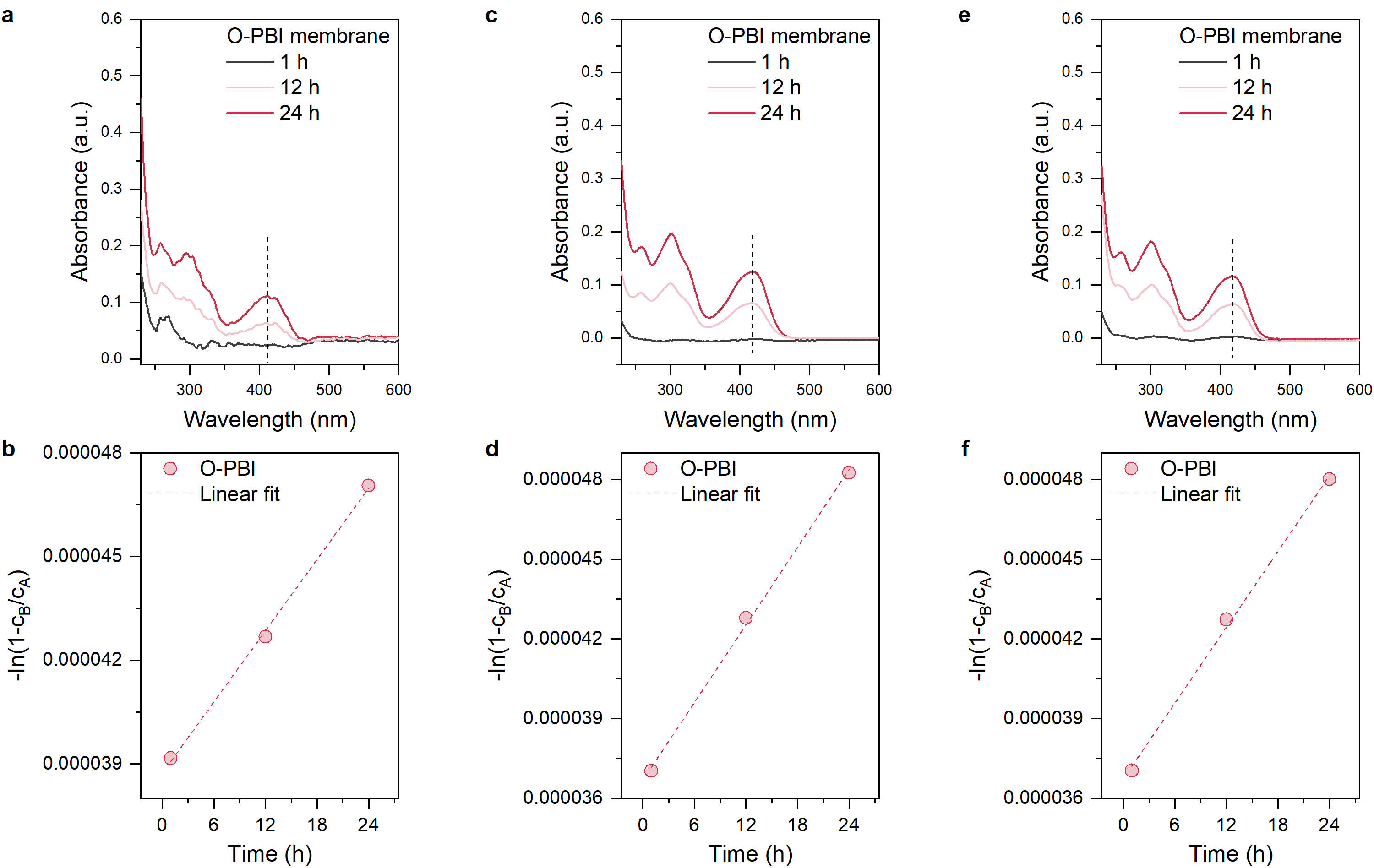


Figure S12. Three sets of the [Fe(CN_6_)]^3−^ permeation through the O-PBI membrane over time. (a, c, e) UV-vis absorption spectra of the permeate side. (b, d, f) -ln(1-c_B_/c_A_) *vs*. permeation time for the determination of permeability of [Fe(CN_6_)]^3−^ under the O-PBI membrane.

.


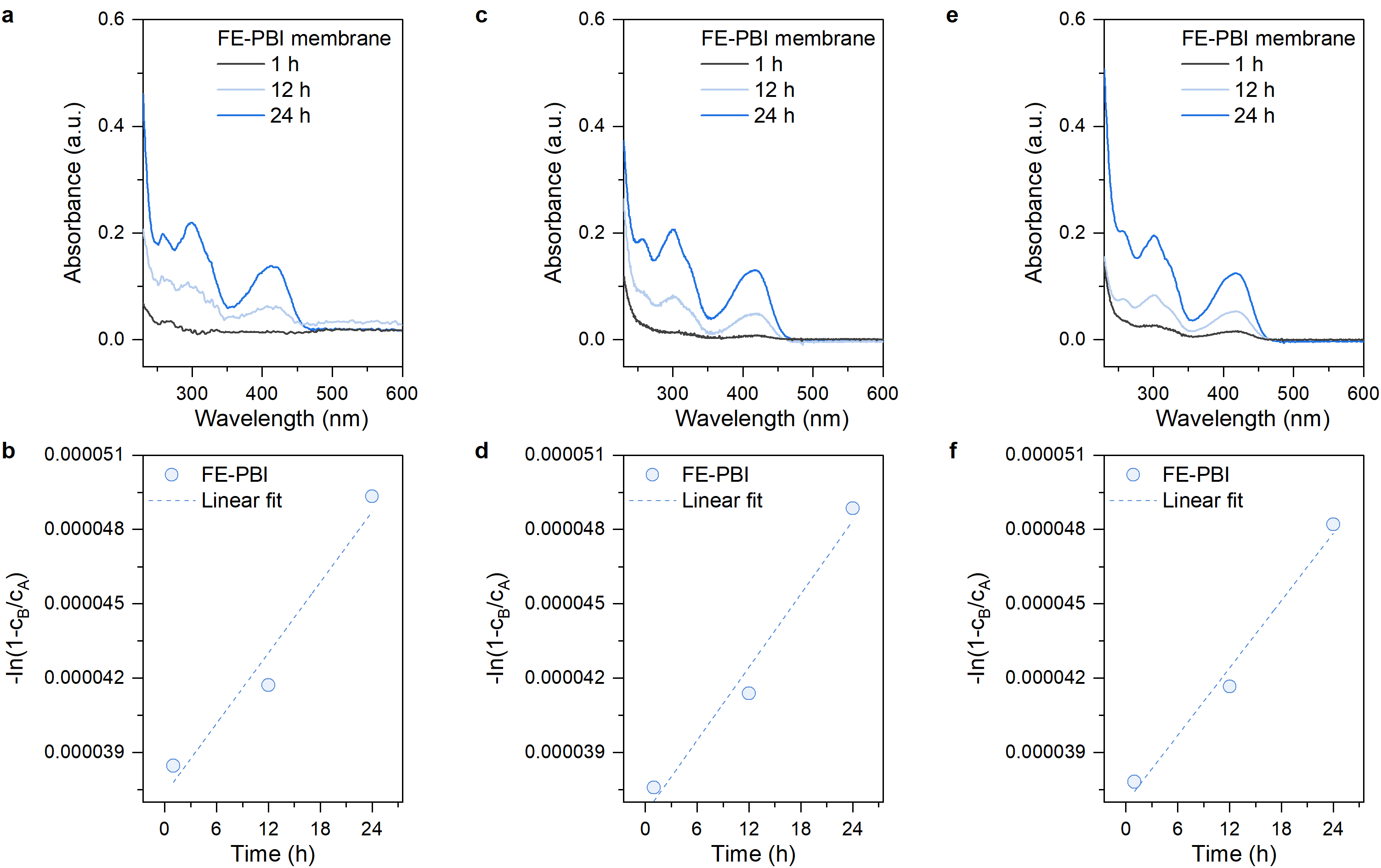


Figure S13. Three sets of the [Fe(CN_6_)]^3−^ permeation through FE-PBI membrane over time. (a, c, e) UV-vis absorption spectra of the permeate side. (b, d, f) -ln(1-c_B_/c_A_) *vs*. permeation time for the determination of permeability of [Fe(CN_6_)]^3−^ under the FE-PBI membrane.


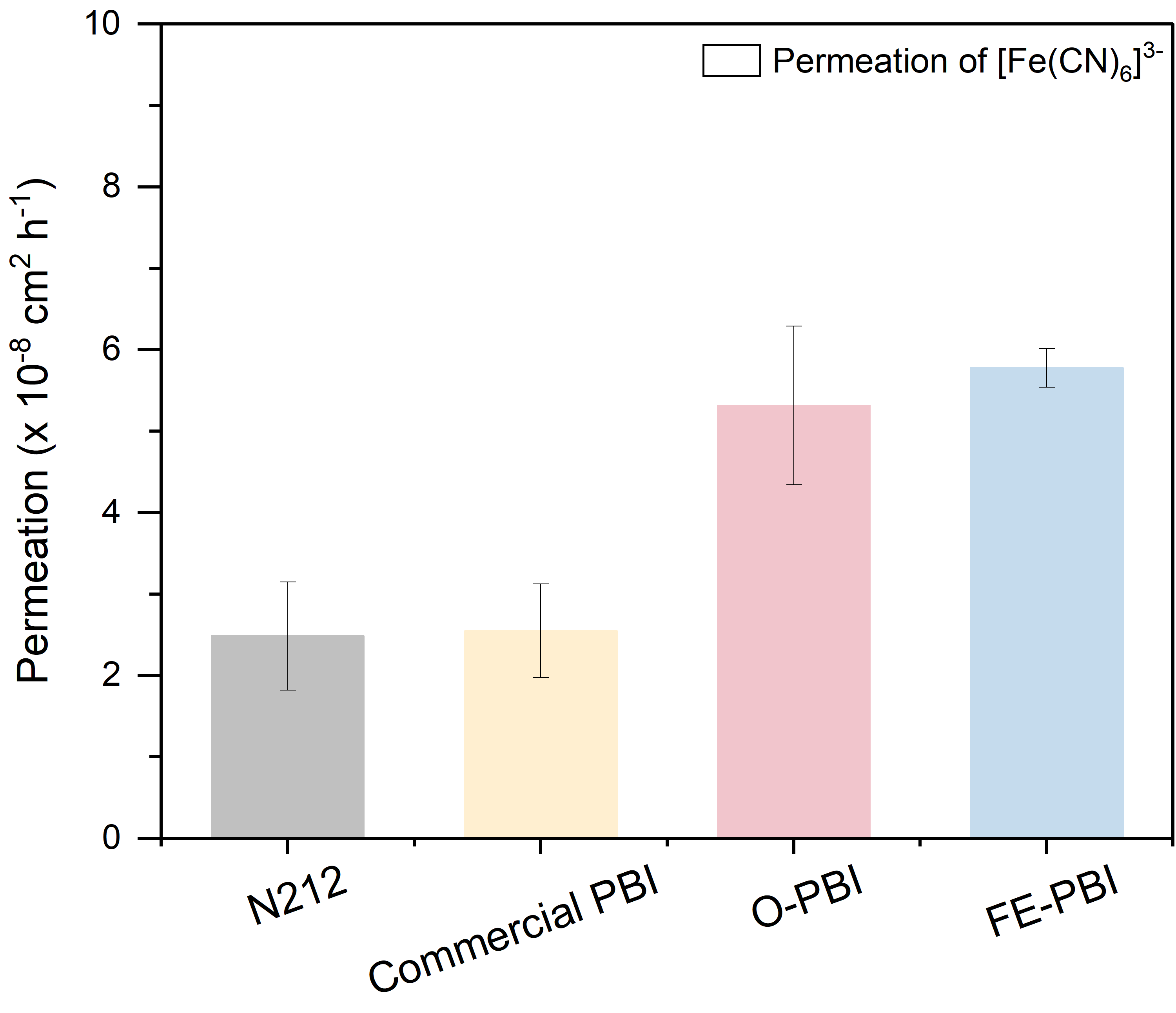


**Figure S14.** Quantitative comparison of permeation across different membranes.


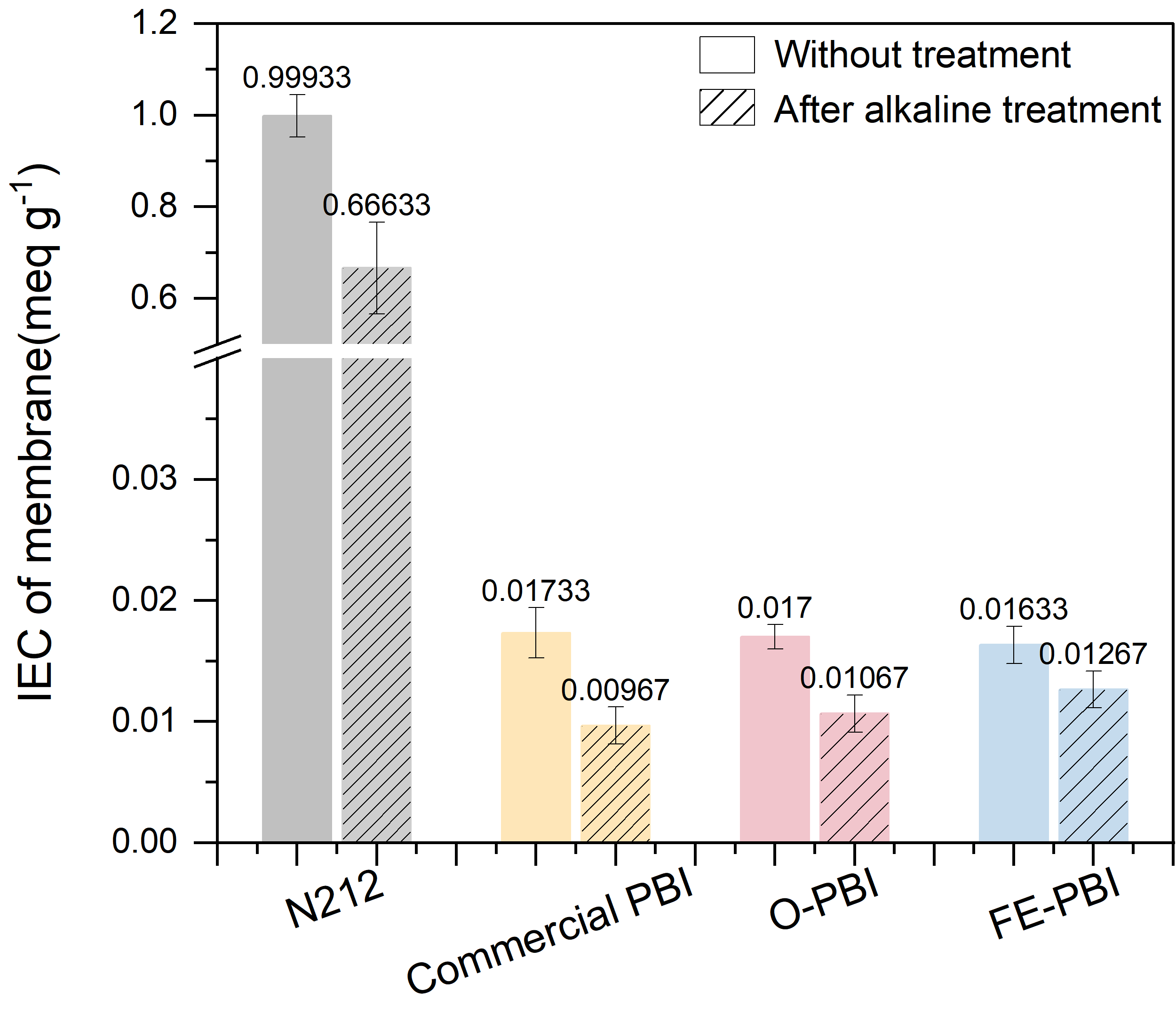


**Figure S15.** IEC of membranes before and after alkaline treatments.


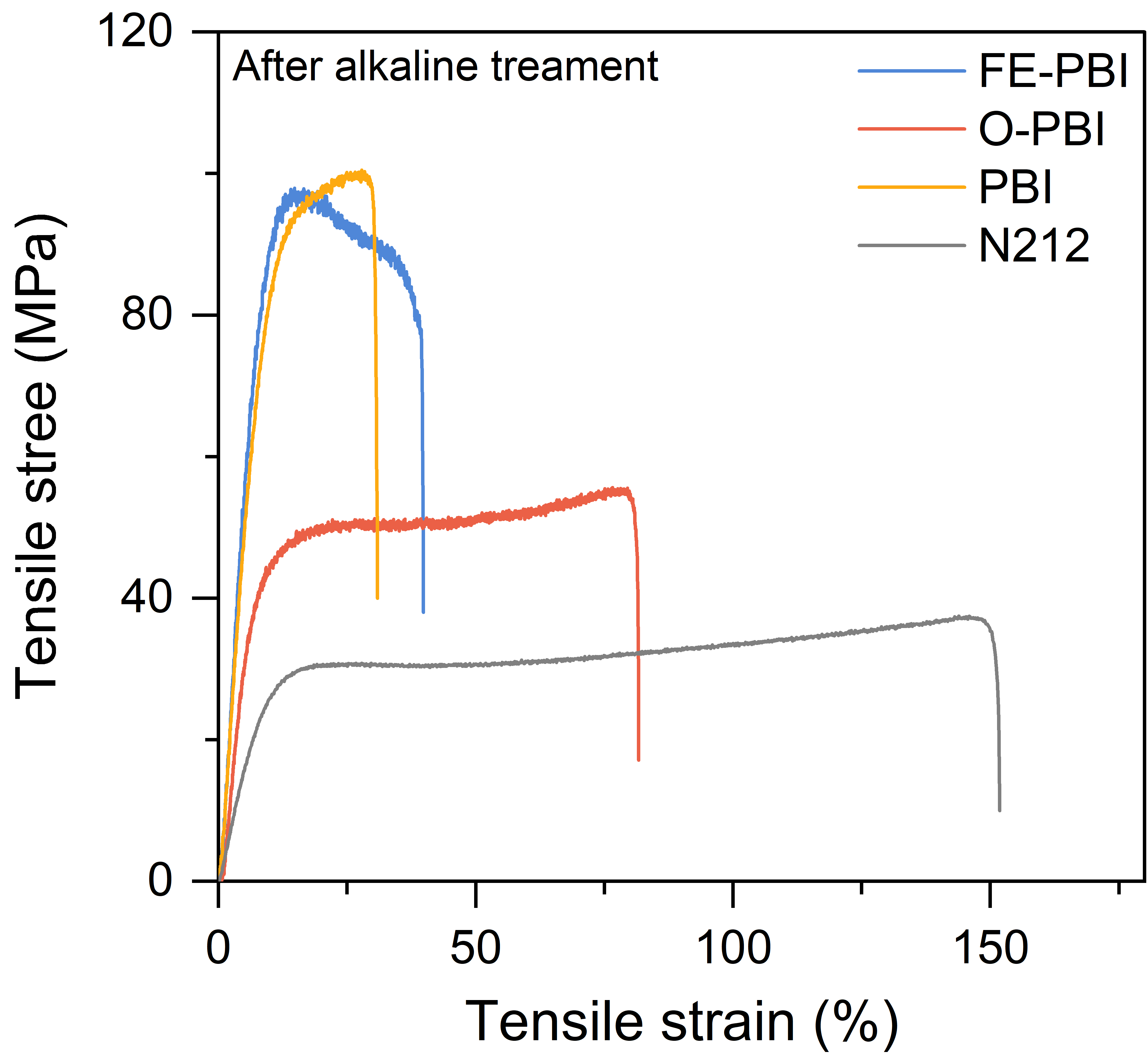


Figure S16. Stress-strain curves of membranes after alkaline treatments.

Table S1. The key parameters and corresponding values for ranking of properties of N212, commercial PBI, O-PBI, and FE-PBI membranes.

| ***Parameters*** | ***N212***  ***Membrane*** | ***Commercial PBI***  ***Membrane*** | ***O-PBI***  ***Membrane*** | ***FE-PBI***  ***Membrane*** |
| --- | --- | --- | --- | --- |
| ***Ionic conductivity*** | 2  (2.57 mS cm^−1^ in KOH) | 2  (3.14 mS cm^−1^ in KOH) | 3  (5.56 mS cm^−1^ in KOH) | 4  (13.42 mS cm^−1^ in KOH) |
| ***Rigidity***  ***(after alkaline treatment)*** | 1  (37.47 MPa) | 4  (24.74 MPa) | 2  (30.62 MPa) | 4  (25.76 MPa) |
| ***Alkaline stability*** | 4  (2.35 mS cm^−1^ in KOH) | 3  (2.33 mS cm^−1^ in KOH) | 1  (2.13 mS cm^−1^ in KOH) | 4  (12.12 mS cm^−1^ in KOH) |
| ***Sustainability*** | 4 | 4 | 4 | 1 |
| ***Safety*** | 2 | 4 | 4 | 4 |
| ***Cost-effectiveness*** | 1  ($ 492.31 m^−2^) | 4.5  (≤ $50 m^−2^) | 4.5  (≤ $50 m^−2^) | 4  (≤ $ 50 m^−2^) |

**Note:** The ionic conductivity, tensile strength, sustainability, safety, and cost-effectiveness were ranked according to the experimental results. The ionic conductivity and alkaline stability were higher than those of membranes (**Figure 2**). The tensile strength was similar for all membranes after alkaline treatment. The parameters of sustainability, safety, and processability were ranked according to the membrane fabrication process. These parameters of N212 membranes were lower than those of PBI-based membranes, which can be attributed to growing concerns about the environmental impacts of Nafion manufacturing and the by-products perfluoroalkyl and polyfluoroalkyl substances (PFAS). Those can be known as “forever chemicals”, which can cause contamination of drinking water and pose long-term threats to human health (https://echa.europa.eu/hot-topics/perfluoroalkyl-chemicals-pfas). Thus, we rank the sustainability, safety, and processability of the Nafion membrane at low values. For better comparison, each parameter was ranked on a scale from 1 to 5 and was qualitative.

**
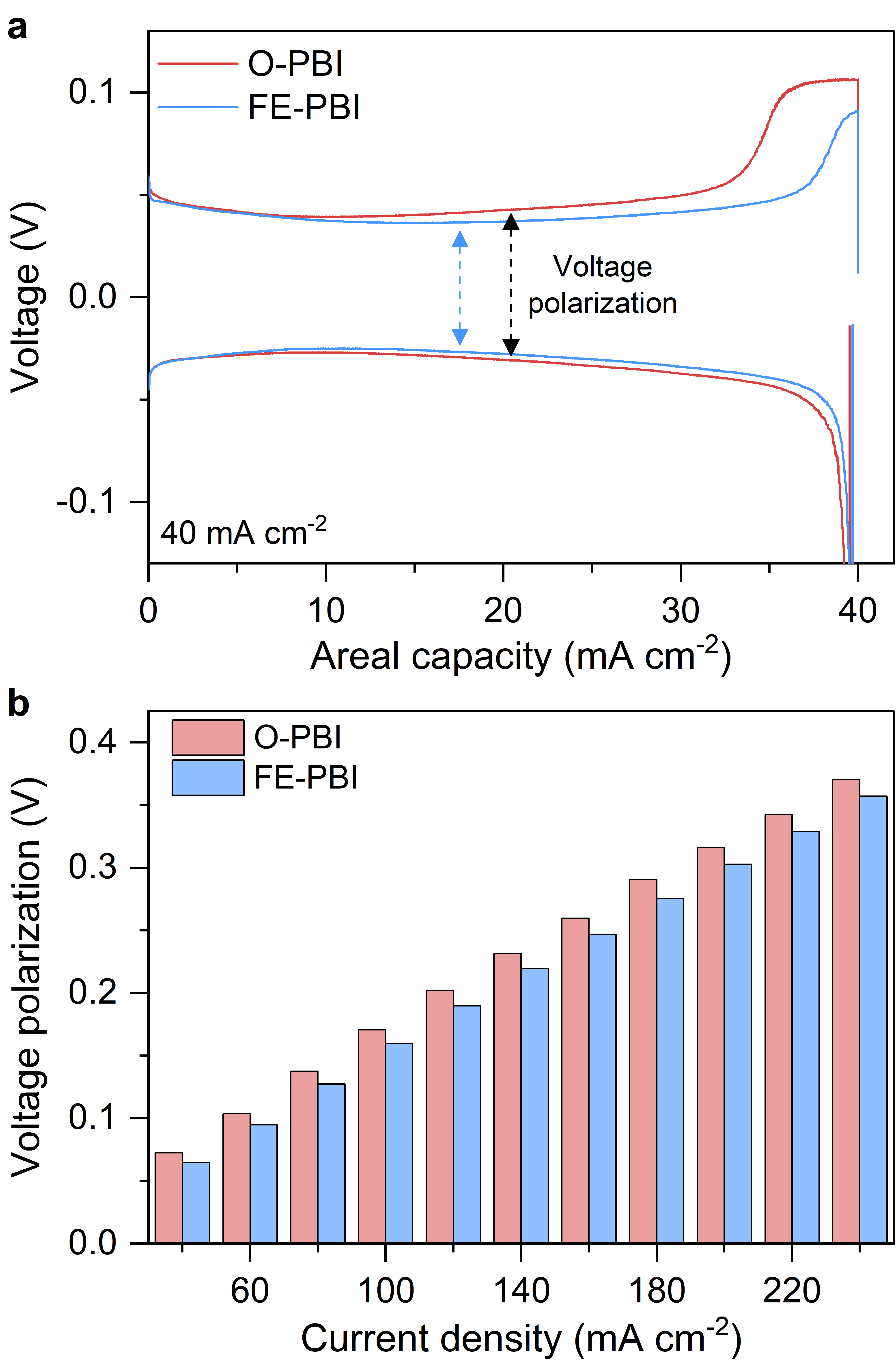
**

Figure S17. Polarization comparison of Zn-based asymmetrical FBs after Zn plating with commercial O-PBI and FE-PBI membranes at different current densities.

**
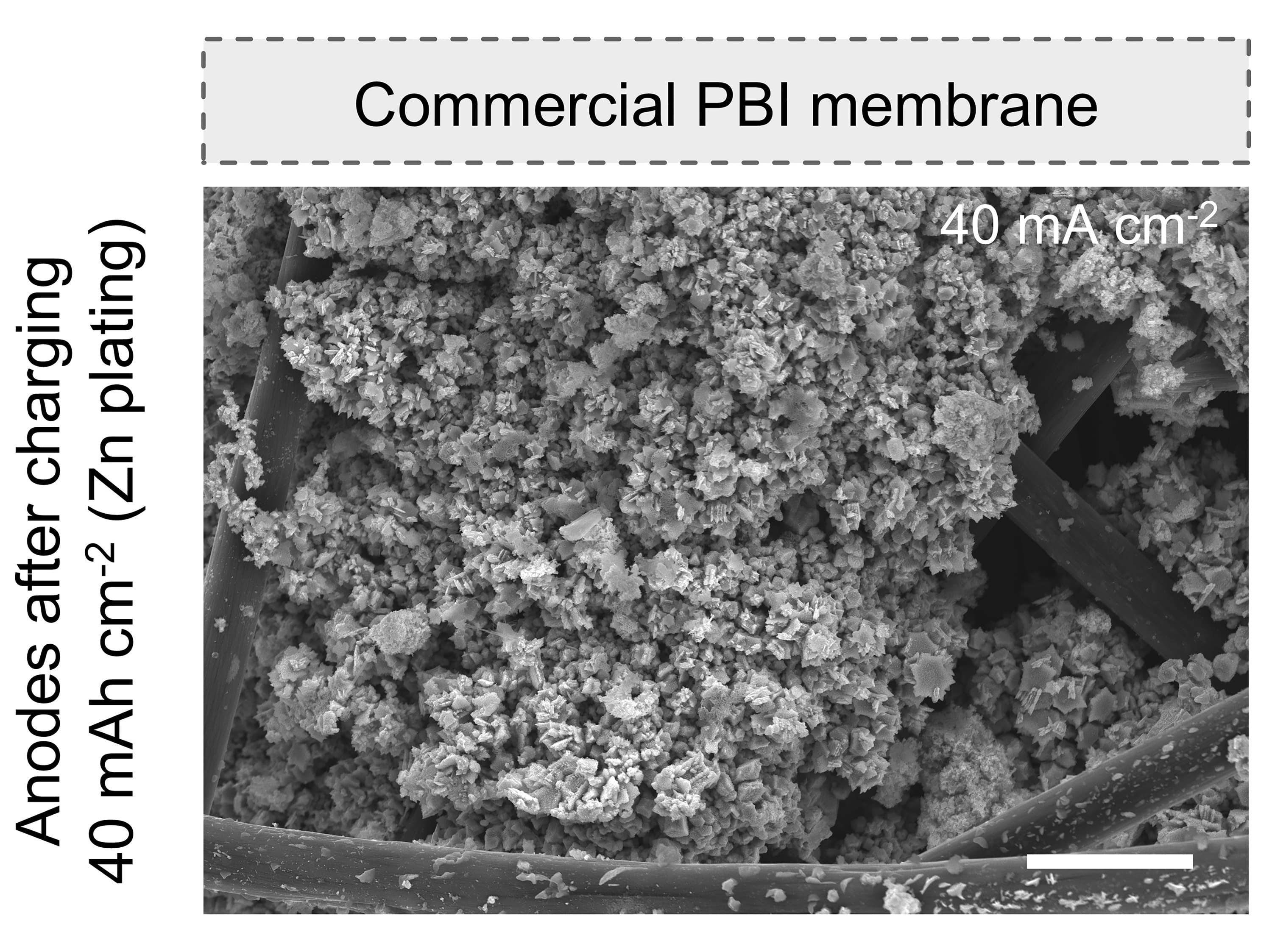
**

Figure S18. **S**EM images of anodes after charging (Zn plating) 40 mAh cm^−2^ at a current density of 40 mA cm^−2^ in alkaline Zn-based asymmetric FBs assembled with commercial PBI membranes.


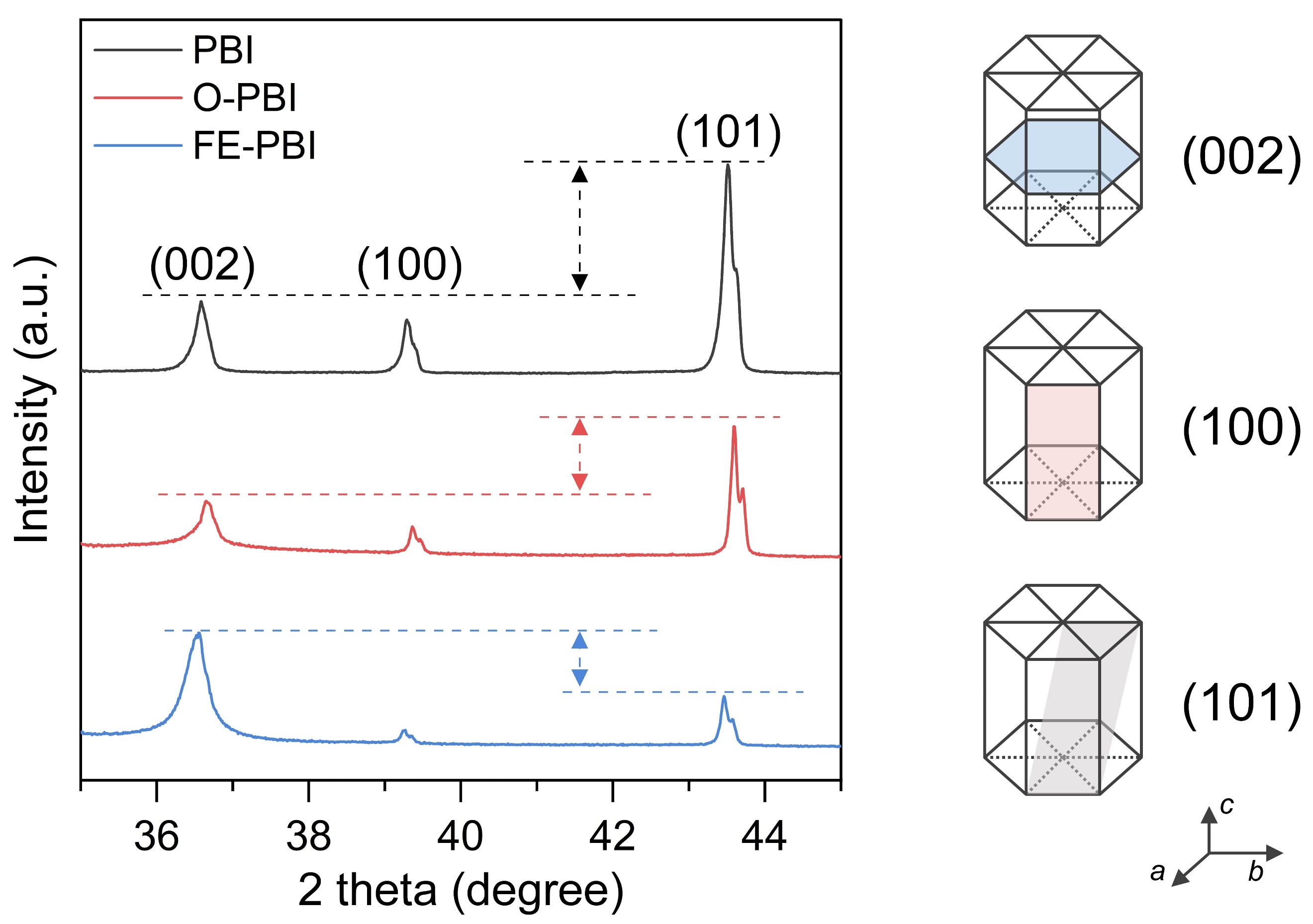


Figure S19. XRD patterns of Zn-based asymmetrical FBs after Zn plating with commercial PBI, O-PBI, and FE-PBI membranes. The right panels corresponded to the relevant crystal planes of Zn metal.


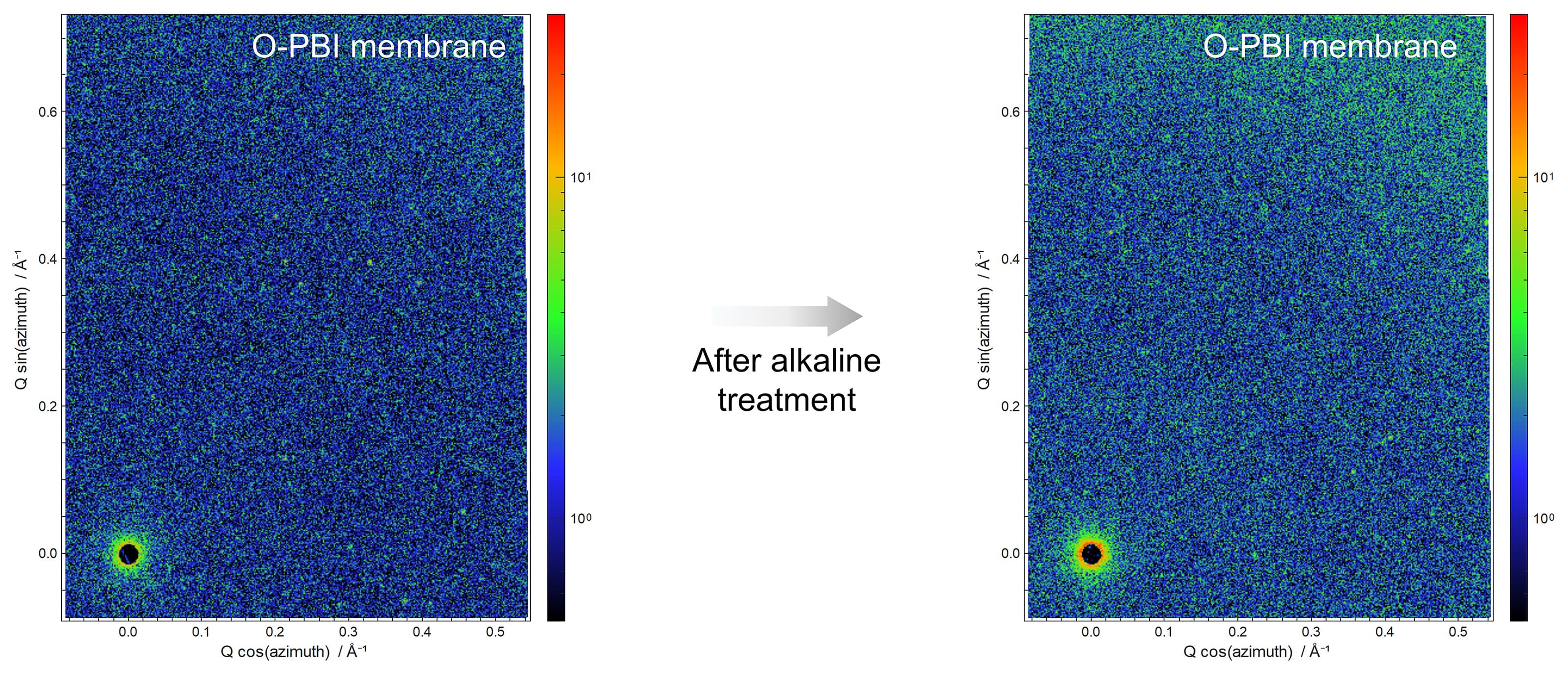


Figure S20. Two-dimensional scattering pattern of O-PBI membranes before/after alkaline treatment.


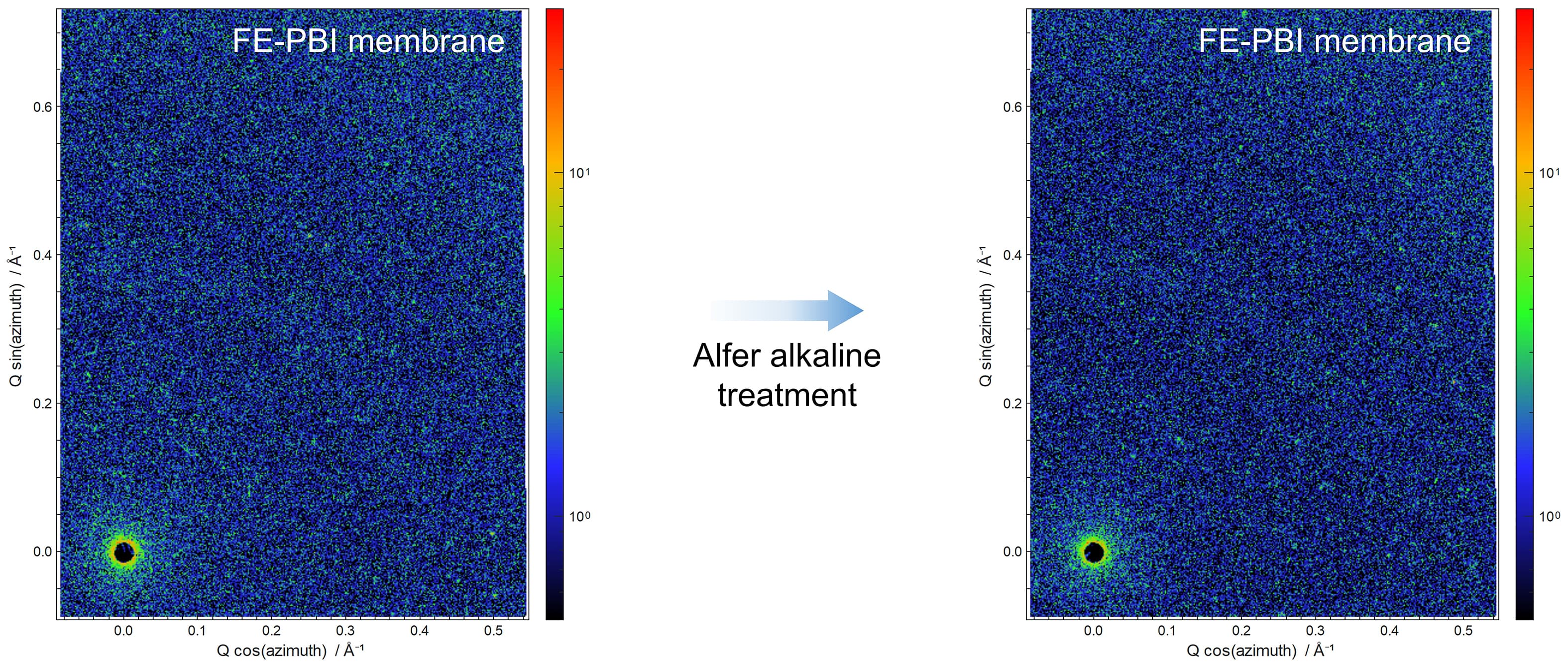


Figure S21. Two-dimensional scattering pattern of FE-PBI membranes before/after alkaline treatment.


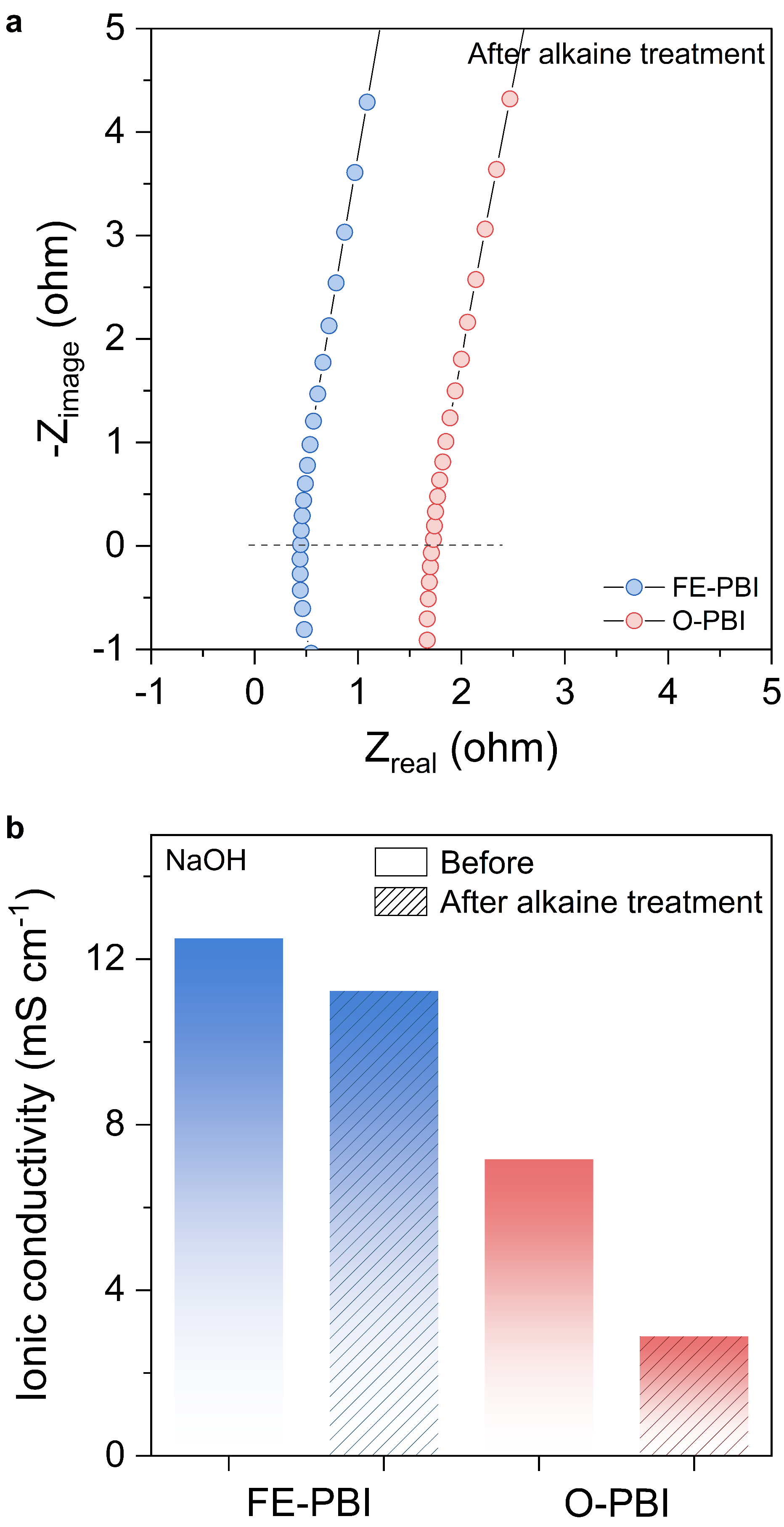


Figure S22. Nyquist plots of O-PBI and FE-PBI membranes before/after alkaline treatment.


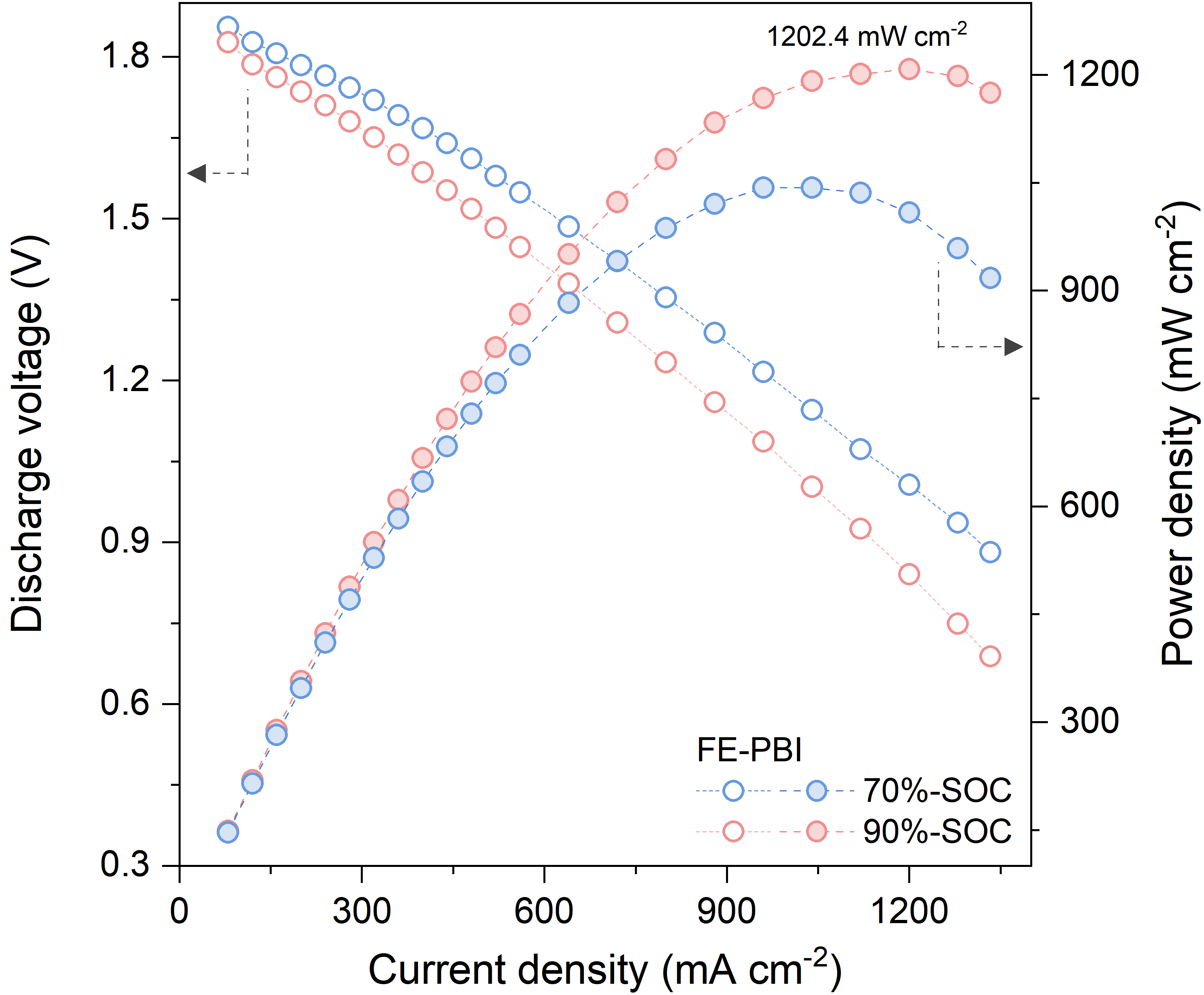


Figure S23. Voltage and power density versus current density of alkaline Zn-Fe FBs cells assembled with FE-PBI membranes at 70% and 90% SOC.


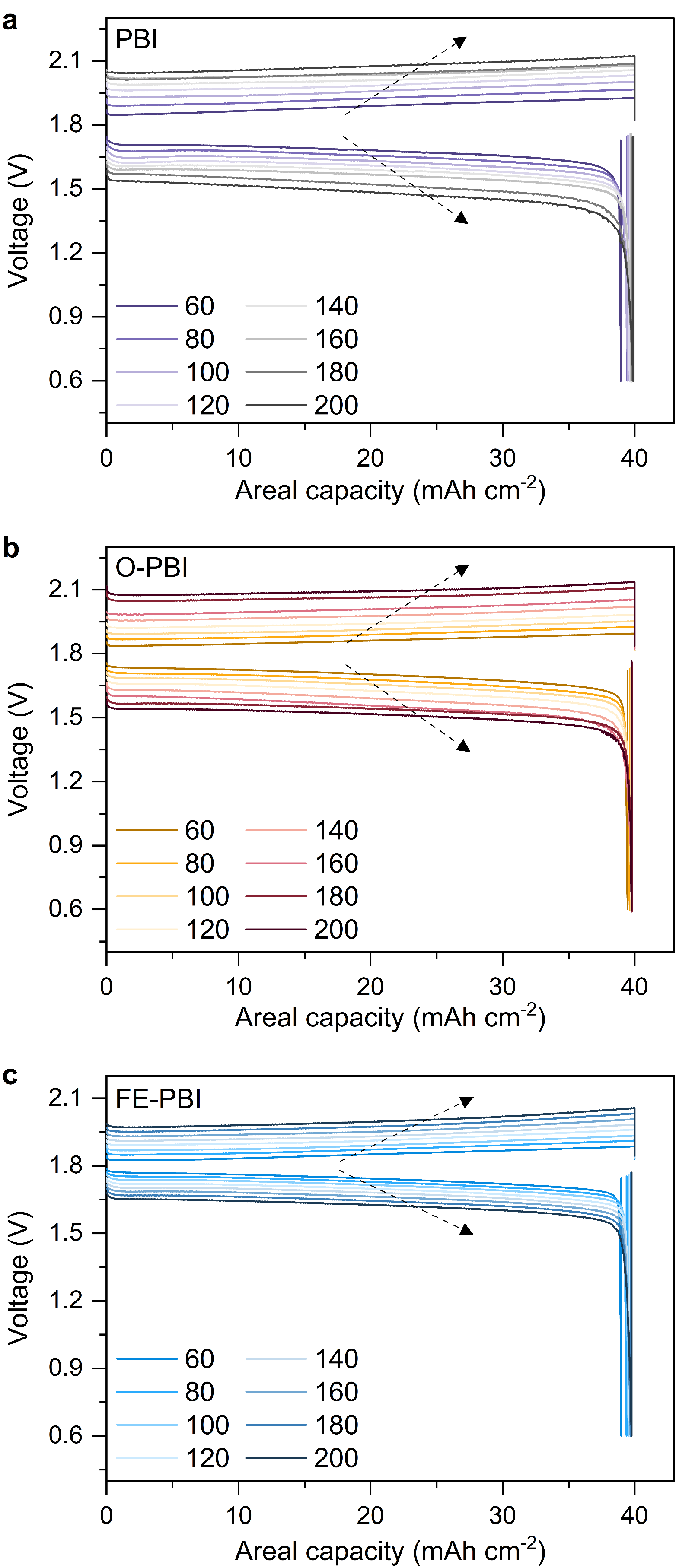


Figure S24. GCD-rate profiles of alkaline Zn-Fe FBs with commercial PBI, O-PBI, and FE-PBI membranes at an areal capacity of 40 mAh cm^−2^ with a current density of 60-200 mA cm^−2^.


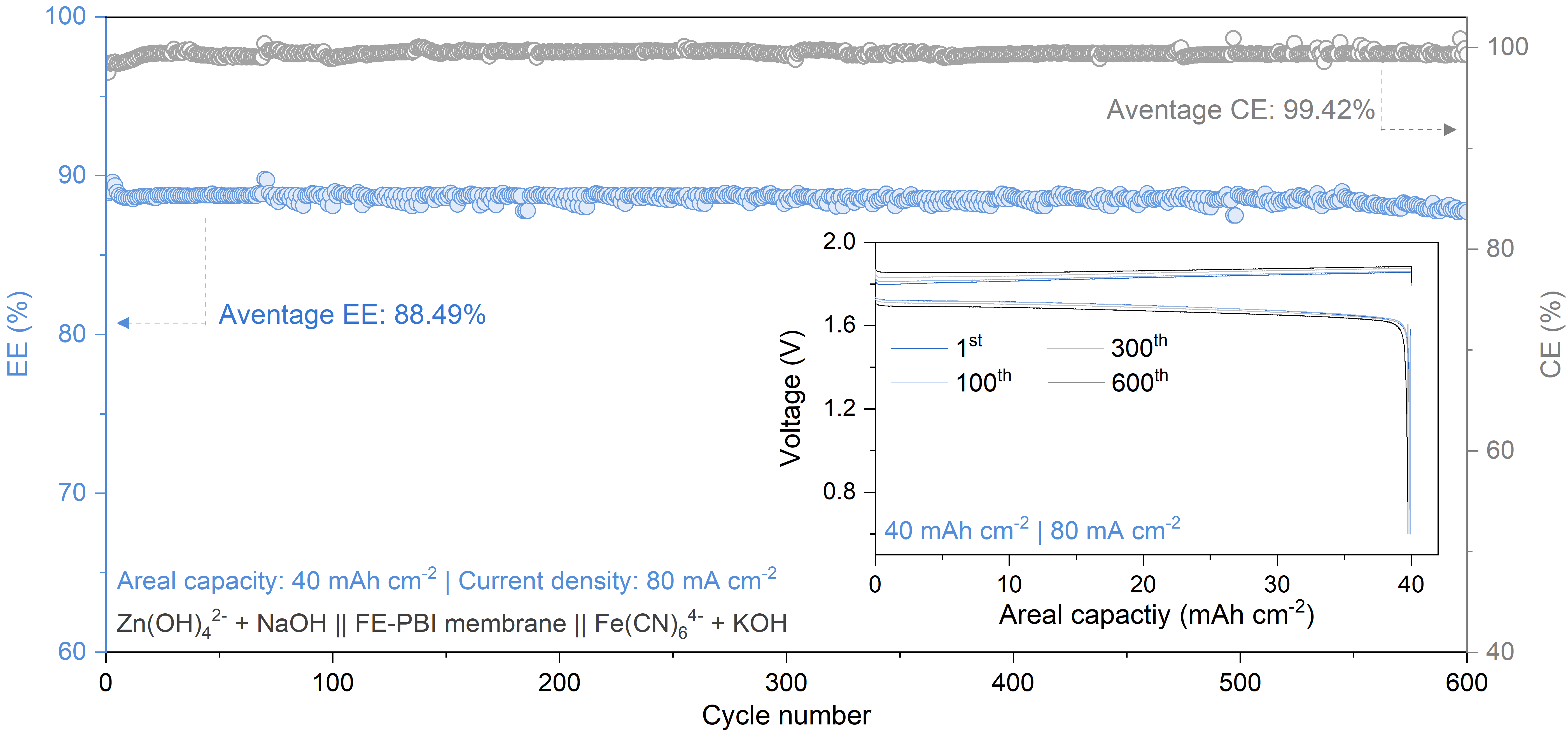


Figure S25. Cycling performance of alkaline Zn-Fe FBs with FE-PBI membranes at an areal capacity of 40 mAh cm^−2^ and current density of 80 mA cm^−2^.


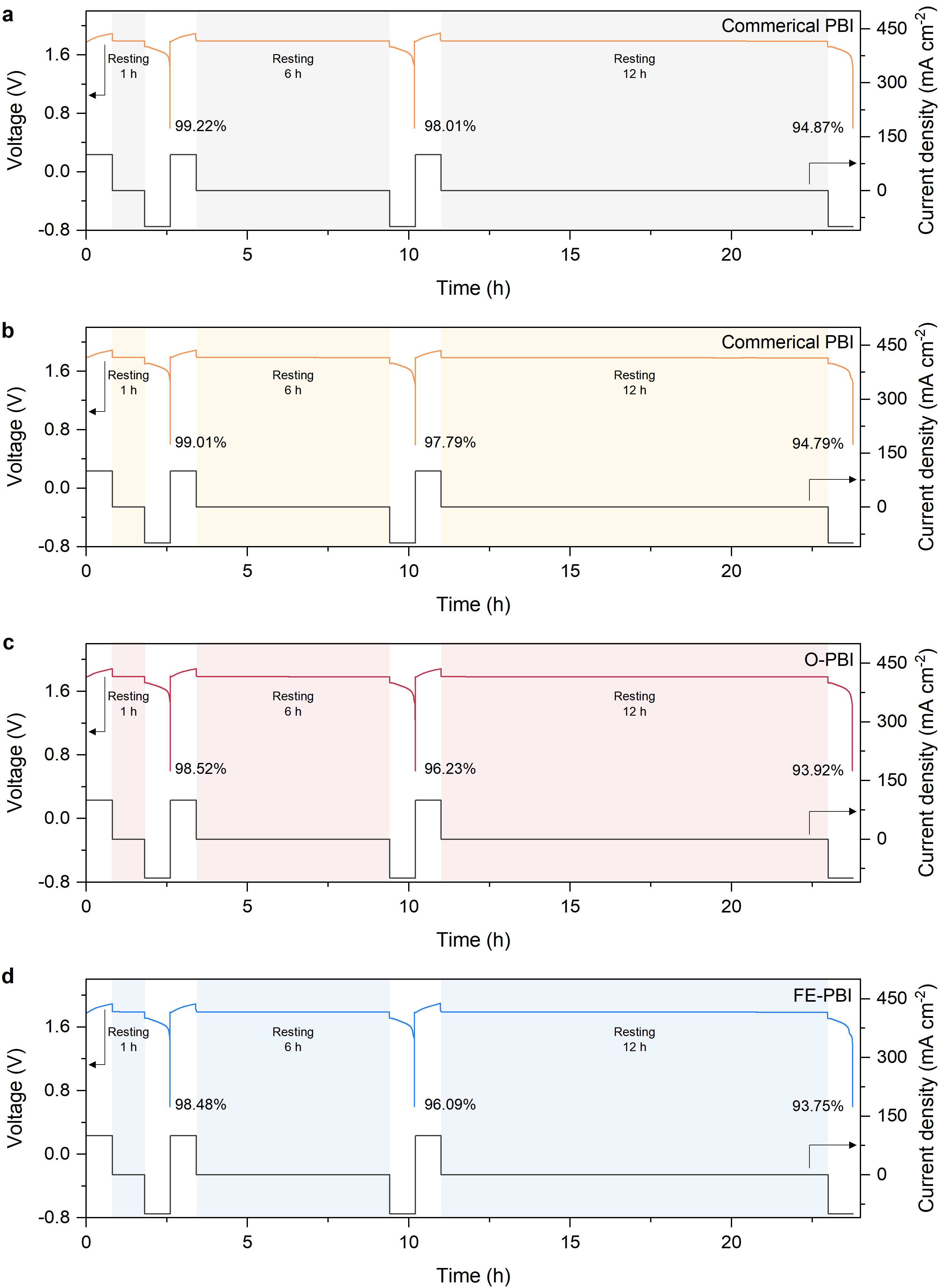


Figure S26. Self-discharge behavior of Zn-Fe FBs with different membranes during resting periods of 1h, 6h, and 12h.

Note: To further connect these permeability results with actual crossover in operating cells, we carried out charged-state resting tests by recording the OCV decay and the retained discharge capacity after resting for 1 h, 6 h, and 12 h (**Figure S26**). As above proved from the permeation results, all FBs exhibit gradual decreases in OCV and retained discharge capacity with increasing resting time. Notably, the FE-PBI-based FBs show self-discharge behavior comparable to that of O-PBI and the other reference membranes, which is consistent with the comparable ferricyanide permeation results. Therefore, the superior Zn–Fe FBs performance of FE-PBI is more reasonably attributed to its intrinsically rigid polymer backbone, which enables a more stable membrane structure and more persistent ion-transport characteristics under alkaline operating conditions.


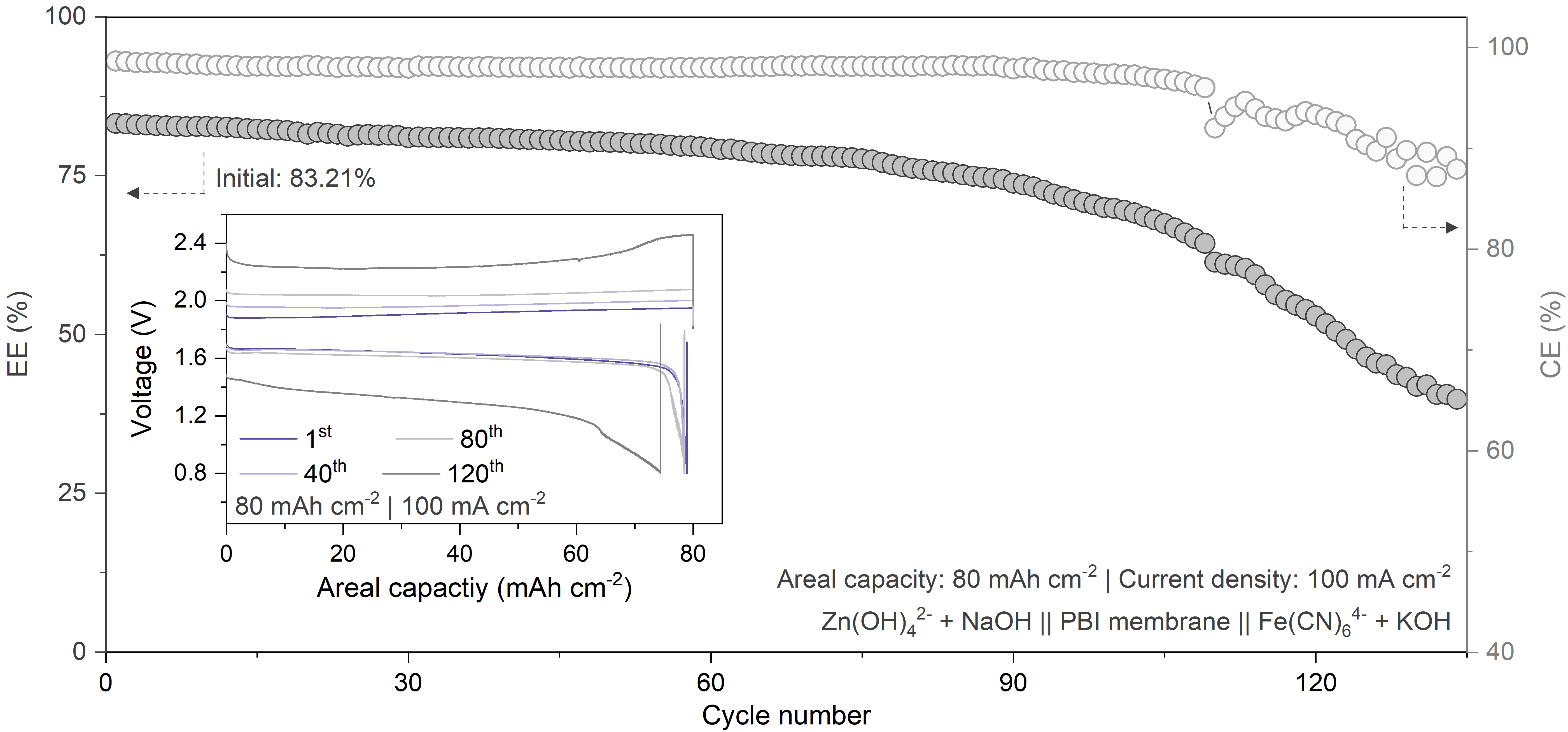


Figure S27. Cycling performance of alkaline Zn-Fe FBs with commercial PBI membranes at an areal capacity of 80 mAh cm^−2^ and current density of 100 mA cm^−2^.


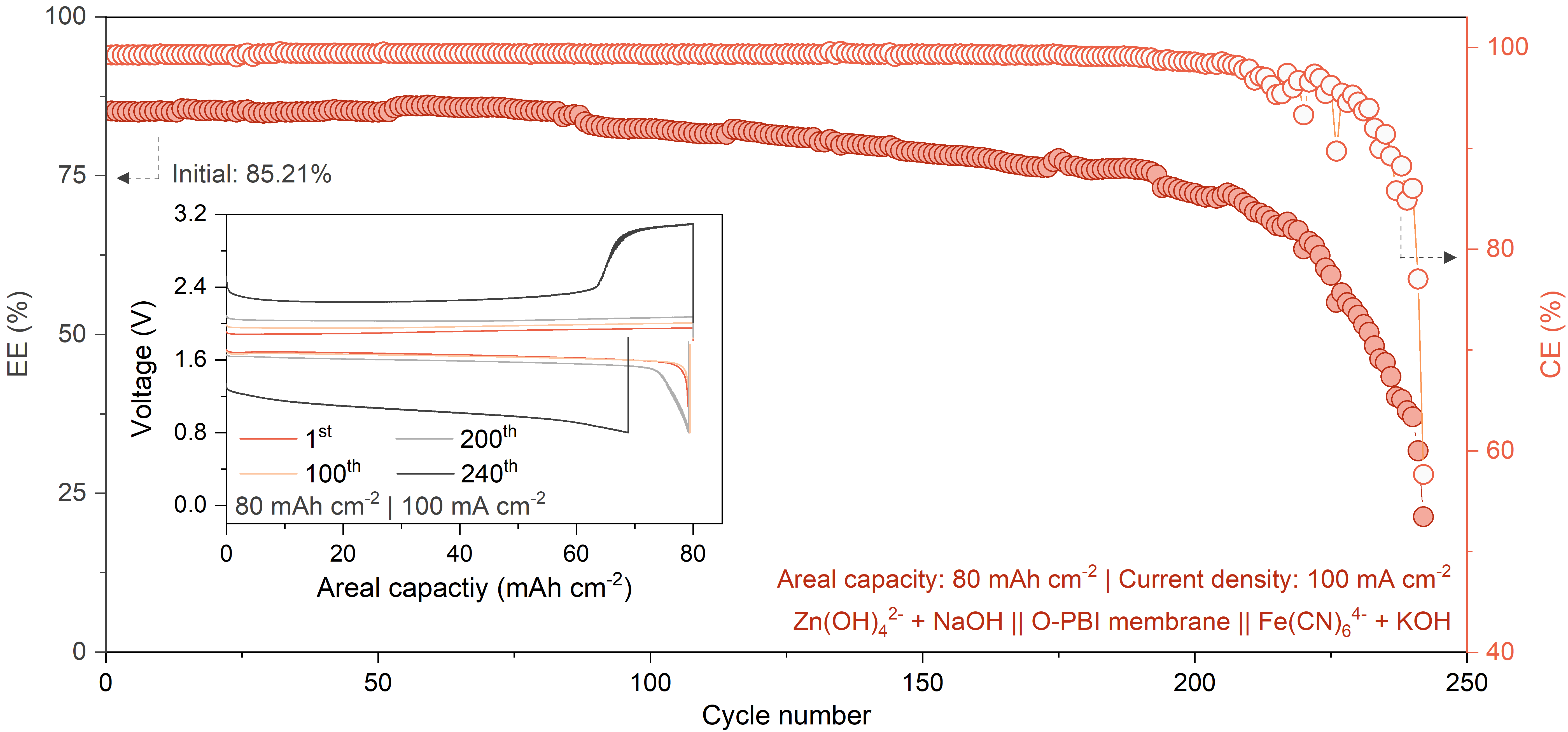


# **Figure S28.** Cycling performance of alkaline Zn-Fe FBs with O-PBI membranes at an areal capacity of 80 mAh cm^−2^ and current density of 100 mA cm^−2^.


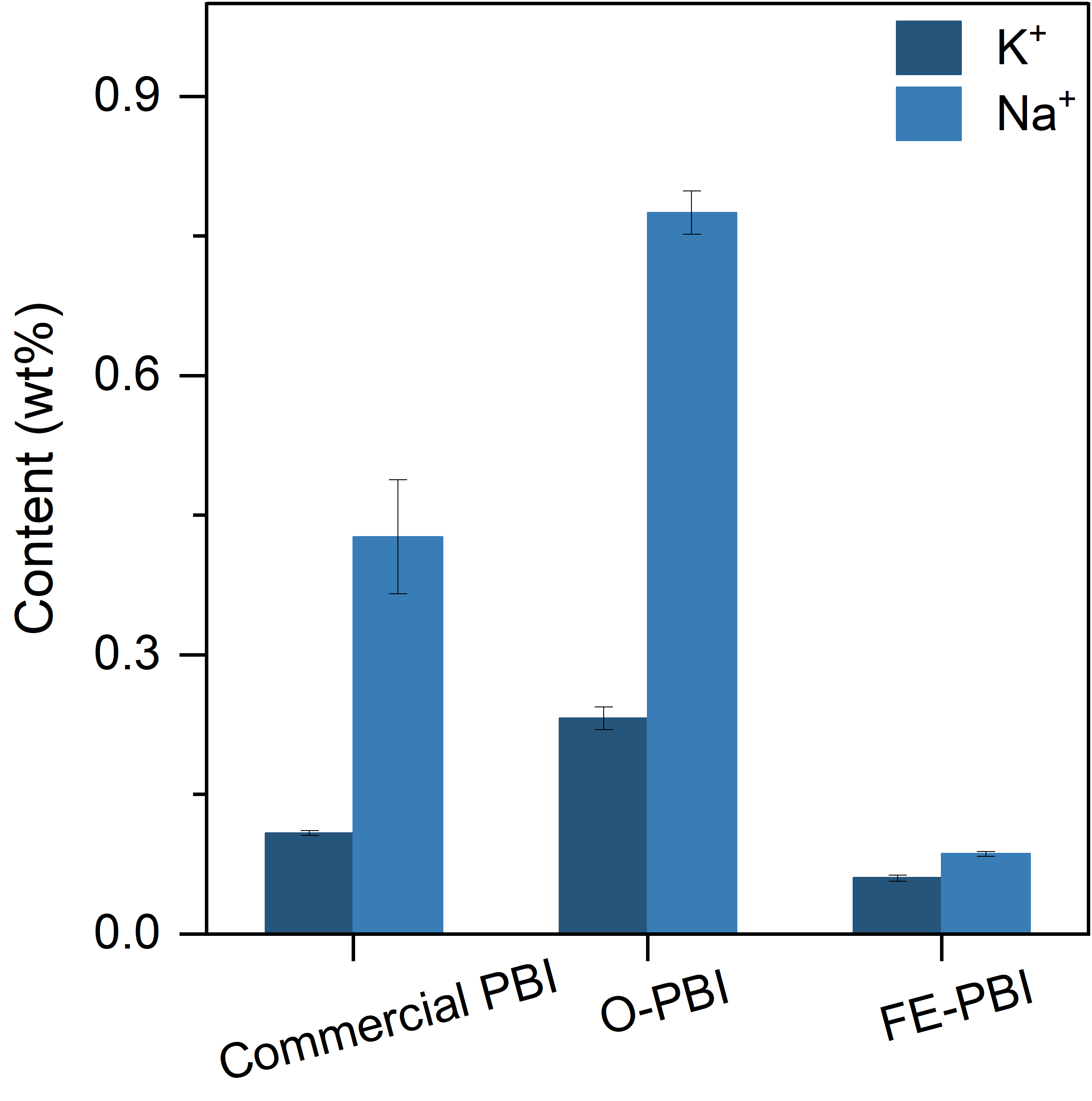


**Figure S29.** K⁺ and Na⁺ content in different membranes after cycles measured by ICP-OES.

**Note:** We further analyzed the Na/K contents in the membranes after cycling using ICP measurements to evaluate alkali-cation interdiffusion and retention under realistic cell conditions. The post-cycling ICP results confirm the occurrence of Na^+^/K^+^ interdiffusion, while FE-PBI exhibits lower alkali-cation retention than O-PBI and pristine PBI (**Figure S29**). This result suggests that the intrinsically rigid FE-PBI backbone is more effective in preserving a more open and continuous ion-transport pathway during cycling. By contrast, the less stable and more flexible polymer frameworks, especially in O-PBI, are more susceptible to structural rearrangement during prolonged operation, which is consistent with increased interlocking/inter-stacking of the ion-transport channels and, consequently, stronger alkali-cation retention within the membrane.


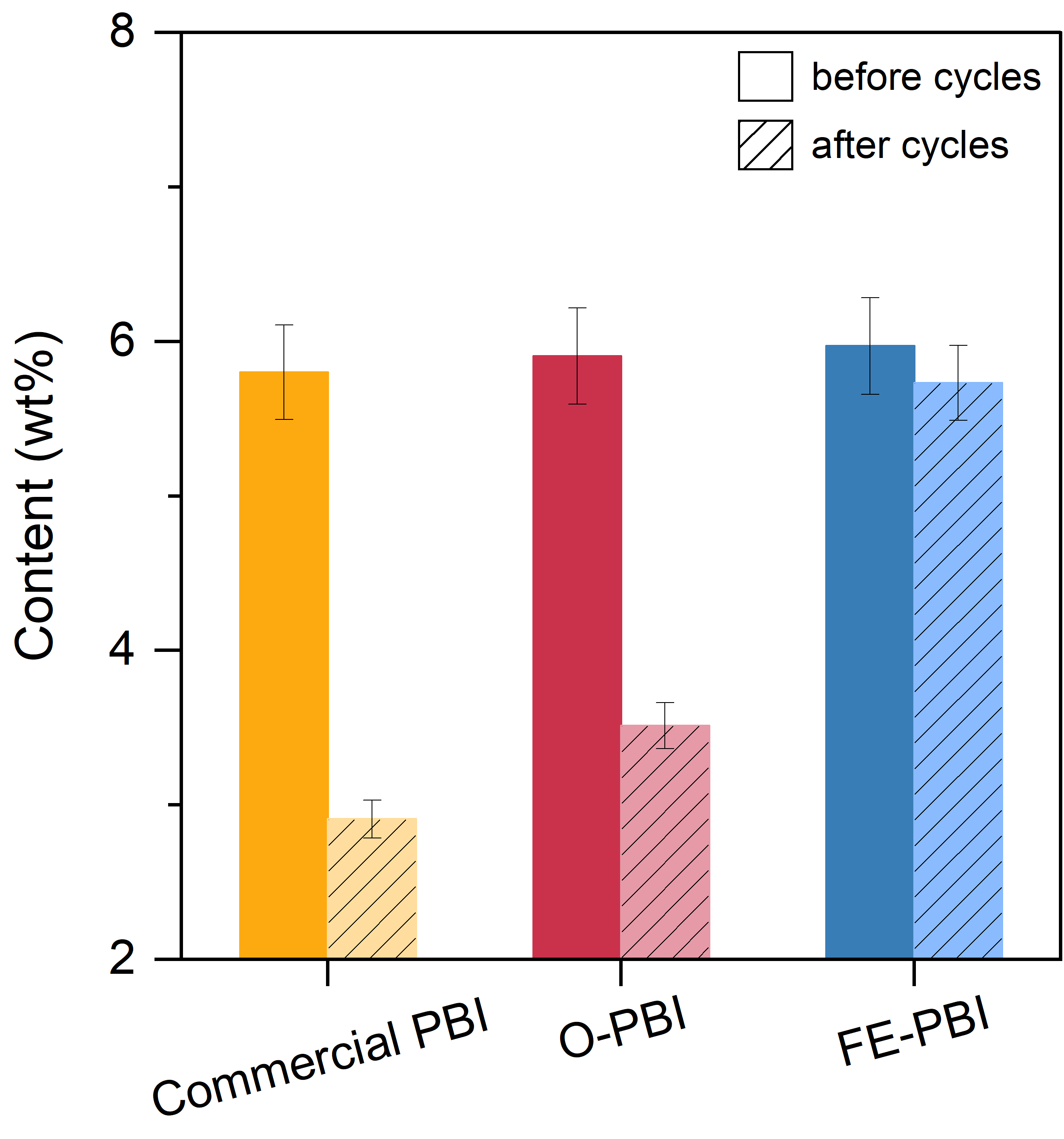


**Figure S30.** P element content in different membranes before/after cycles measured by ICP-OES.

**Note:** We further quantified the phosphorus (P) contents of the membranes after washing/cycling by ICP to demonstrate the structural stability of the polymer framework through the evolution of phosphorus content (H_3_PO_4_). Firstly, a less structurally stable membrane may be accompanied by a greater loss of phosphorus-containing species, which can be attributed to more pronounced structural rearrangement or collapse during cycling. By contrast, a structurally more robust membrane could exhibit a more controlled evolution of phosphorus content, together with better retention of ion-transport properties. As shown in **Figure S30**, the phosphorus contents of O-PBI and PBI change much more substantially before and after cycling, whereas FE-PBI shows only a limited variation in phosphorus content, which can be attributed to the superior stability of its membrane backbone. These results indicate that the enhanced membrane performance of FE-PBI is more closely associated with the intrinsic structural robustness of the fix-linkage-based polymer framework, which is well aligned with other results in our manuscripts.

Table S2. Comparison of Alkaline Zn-Fe FBs performance between this work and previously reported work.

|  | ***System*** | ***Current density***  ***(mA cm*^−^*^2^)*** | ***Areal capacity***  ***(mAh cm*^−^*^2^)*** | ***Cycle number*** | ***Lifespan***  ***(hour)*** | ***Energy Efficiency (%)*** | ***Reference*** |
| --- | --- | --- | --- | --- | --- | --- | --- |
| *1* | *Membrane* | *80* | *100* | *140* | *340* | *86.7* | *Nat Commun*, 2023, **14**, 1149 |
| *2* | *Membrane* | *80* | *40* | *-* | *300* | *90.3* | *Adv. Energy Mater.* 2023, **13**, 2300779 |
| *3* | *Additives* | *50* | *30* | *150* | *120* | *89.1* | *Adv. Mater.* 2024, 2404834 |
| *4* | *Additives* | *80* | *100* | *500* | *350* | *85.8* | *Energy Environ. Sci.*, 2024, **17**, 717 |
| *5* | *Electrode* | *880* | *-* | *225* | *-* | *89.7* | *Small* 2024, **20**, 2308791 |
| *6* | *Electrolytes* | *40* | *-* | *200* | *-* | *86* | *Angew. Chem.Int. Ed.* 2023, **62**, e202304667 |
| *7* | *Membrane* | *80* | *66* | *220* | *350* | *88.01* | *Adv. Funct. Mater.* 2023, **33**, 2301448 |
| *8* | *Membrane* | *80* | *20* | *400* | *100* | *80* | *Joule*, 2022, **6**, 884 |
| *9* | *Membrane* | *80* | *160* | *400* | *100* | *90.1* | *J. Am. Chem. Soc.* 2021, **143**, 13135 |
| ***This***  ***work*** | *Membrane* | ***80*** | ***40*** | ***600*** | ***600*** | ***88.49*** | ***-*** |
| ***This***  ***work*** | *Membrane* | ***100*** | ***80*** | ***500*** | ***800*** | ***85.56*** | ***-*** |

# Reference

1. Maurya, S. et al. Phosphoric acid pre-treatment to tailor polybenzimidazole membranes for vanadium redox flow batteries. *J. Membr. Sci.* **668**, 121233 (2023).

2. Tan, R. et al. Hydrophilic microporous membranes for selective ion separation and flow-battery energy storage. *Nat Mater* **19**, 195-202 (2020).

3. Huang, X. et al. Engineered PES/SPES nanochannel membrane for salinity gradient power generation. *Nano Energy* **59**, 354-362 (2019).

4. Chen, J. et al. Ultrathin and robust silk fibroin membrane for high-performance osmotic energy conversion. *ACS Energy Lett.* **5**, 742-748 (2019).

5. Yuan, Z. et al. Low-cost hydrocarbon membrane enables commercial-scale flow batteries for long-duration energy storage. *Joule* **6**, 884-905 (2022).

6. He, S. S. & Frank, C. W. Facilitating hydroxide transport in anion exchange membranes via hydrophilic grafts. *J. Mater. Chem. A* **2**, 16489-16497 (2014).

7. Ren, C. E. et al. Charge-and size-selective ion sieving through Ti3C2T x MXene membranes. *J. Phys. Chem. Lett.* **6**, 4026-4031 (2015).

8. Haynes, W. M. *CRC handbook of chemistry and physics*. (CRC press, 2016).

9. Li, Z. & Lu, Y.-C. Polysulfide-based redox flow batteries with long life and low levelized cost enabled by charge-reinforced ion-selective membranes. *Nat. Energy* **6**, 517-528 (2021).

10. Frisch, M. et al. Gaussian 16 Rev. A. 03.; Wallingford CT (2016) GaussView 5.0.; Gaussian. *Inc.: Wallingford, UK* (2016).

11. Zhao, Y. & Truhlar, D. G. Exploring the limit of accuracy of the global hybrid meta density functional for main-group thermochemistry, kinetics, and noncovalent interactions. *Journal of Chemical Theory and Computation* **4**, 1849-1868 (2008).

12. Weigend, F. & Ahlrichs, R. Balanced basis sets of split valence, triple zeta valence and quadruple zeta valence quality for H to Rn: Design and assessment of accuracy. *Physical Chemistry Chemical Physics* **7**, 3297-3305 (2005).

13. Grimme, S. Density functional theory with London dispersion corrections. *Wiley Interdisciplinary Reviews: Computational Molecular Science* **1**, 211-228 (2011).

14. Grimme, S. Accurate description of van der Waals complexes by density functional theory including empirical corrections. *Journal of computational chemistry* **25**, 1463-1473 (2004).

15. Lu, T. & Chen, F. Multiwfn: A multifunctional wavefunction analyzer. *Journal of computational chemistry* **33**, 580-592 (2012).

16. Humphrey, W., Dalke, A. & Schulten, K. VMD: visual molecular dynamics. *Journal of molecular graphics* **14**, 33-38 (1996).

17. Wu, J. et al. Metal-coordinated polybenzimidazole membranes with preferential K(+) transport. *Nat Commun* **14**, 1149 (2023).

18. Nature CommunicationsZuo, P. et al. Near-frictionless ion transport within triazine framework membranes. *Nature* **617**, 299-305 (2023).
